# LIM and SH3 Protein -1 Modulates CXCR2-Mediated Cell Migration

**DOI:** 10.1371/journal.pone.0010050

**Published:** 2010-04-19

**Authors:** Dayanidhi Raman, Jiqing Sai, Nicole F. Neel, Catherine S. Chew, Ann Richmond

**Affiliations:** 1 Department of Cancer Biology, Vanderbilt University School of Medicine, Nashville, Tennessee, United States of America; 2 Department of Veterans Affairs, Nashville, Tennessee, United States of America; 3 Institute of Molecular Medicine and Genetics, Medical College of Georgia, Augusta, Georgia, United States of America; New York University, United States of America

## Abstract

**Background:**

The chemokine receptor CXCR2 plays a pivotal role in migration of neutrophils, macrophages and endothelial cells, modulating several biological responses such as angiogenesis, wound healing and acute inflammation. CXCR2 is also involved in pathogenesis of chronic inflammation, sepsis and atherosclerosis. The ability of CXCR2 to associate with a variety of proteins dynamically is responsible for its effects on directed cell migration or chemotaxis. The dynamic network of such CXCR2 binding proteins is termed as “CXCR2 chemosynapse”. Proteomic analysis of proteins that co-immunoprecipitated with CXCR2 in neutrophil-like dHL-60 cells revealed a novel protein, LIM and SH3 protein 1 (LASP-1), binds CXCR2 under both basal and ligand activated conditions. LASP-1 is an actin binding cytoskeletal protein, involved in the cell migration.

**Methodology/Principal Findings:**

We demonstrate that CXCR2 and LASP-1 co-immunoprecipitate and co-localize at the leading edge of migrating cells. The LIM domain of LASP-1 directly binds to the carboxy-terminal domain (CTD) of CXCR2. Moreover, LASP-1 also directly binds the CTD of CXCR1, CXCR3 and CXCR4. Using a site-directed and deletion mutagenesis approach, Iso323-Leu324 of the conserved LKIL motif on CXCR2-CTD was identified as the binding site for LASP-1. Interruption of the interaction between CXCR2-CTD and LIM domain of LASP-1 by dominant negative and knock down approaches inhibited CXCR2-mediated chemotaxis. Analysis for the mechanism for inhibition of CXCR2-mediated chemotaxis indicated that LASP-1/CXCR2 interaction is essential for cell motility and focal adhesion turnover involving activation of *Src*, paxillin, PAK1, p130CAS and ERK1/2.

**Conclusions/Significance:**

We demonstrate here for the first time that LASP-1 is a key component of the “CXCR2 chemosynapse” and LASP-1 interaction with CXCR2 is critical for CXCR2-mediated chemotaxis. Furthermore, LASP-1 also directly binds the CTD of CXCR1, CXCR3 and CXCR4, suggesting that LASP-1 is a general mediator of CXC chemokine mediated chemotaxis. Thus, LASP-1 may serve as a new link coordinating the flow of information between chemokine receptors and nascent focal adhesions, especially at the leading edge. Thus the association between the chemokine receptors and LASP-1 suggests to the presence of a CXC chemokine receptor-LASP-1 pro-migratory module in cells governing the cell migration.

## Introduction

The chemokine receptor CXCR2 is a major modulator of inflammation, angiogenesis, tumor growth, and wound healing [1]–[8]. Chemotactic migration is a complex process that involves sensing of the chemokine cues in the extracellular environment, polarization of intracellular signaling leading to protrusion of the lamellipodium through actin polymerization, integrin mediated adhesion of the lamellipodium to the substratum (adhesion assembly), cell body translocation, adhesion disassembly and tail retraction [9]–[14]. The CXC chemokine receptor, CXCR2, directs the migration of leukocytes and endothelial cells towards a chemokine gradient. The ability of CXCR2 to bind to a repertoire of proteins during intracellular trafficking dictates its ability to facilitate directed migration of leukocytes and endothelial cells. The dynamic and functional association of proteins with CXCR2 during ligand activation, subsequent signaling, and receptor trafficking establishes a functional ‘chemosynapse’ [15], [16]. For example, CXCR2 interacts with adaptor protein 2 (AP-2), β-arrestin, heat shock protein-70 interacting protein (HIP), Protein Phosphatase 2A, and vasodilator-stimulated phosphoprotein (VASP) [17]–[19]. Proteomic analysis of the proteins that co-immunoprecipitate with CXCR2 in human neutrophil-like dHL-60 cells revealed that CXCR2 binds a novel protein, LIM and SH3 protein 1 (LASP-1), under both basal and ligand activated conditions [15]. LASP-1 has not been previously shown to bind to CXCR2.

LASP-1 is a cytoskeletal scaffold protein originally discovered from the cDNA library of human metastatic breast cancer [20]. LASP-1 is organized into three domains: N-terminal LIM (Lin/Isl/Mec) domain, a domain with two nebulin repeats (NR) and a C-terminal SH3 domain. The N-terminal LIM domain is a protein-protein interaction module that has two zinc fingers separated by two amino acids [21]. Through its NR domain, LASP-1 directly binds to actin *in vitro* with a stoichiometry of 1∶7 [22]. The SH3 domain binds to proteins containing a polyproline motif, including zyxin and the 140 kDa isoform of palladin [23]. Interestingly, zyxin, a LASP-1 binding protein was observed in nascent adhesions at the leading edge of the migratory cells [12]. LASP-1 is reported to be enriched in pseudopodia and to localize to nascent focal complexes [24], [25]. At the tips of the pseudopodia, LASP-1 associates with Kelch related protein 1 (Krp1) in transformed fibroblasts [25]. LASP-1 seems to play a critical role in cytoskeletal organization and cell migration. LASP-1 mediates cell migration, proliferation and survival in several mammary and ovarian carcinoma cell lines. Silencing of LASP-1 inhibits cell migration and proliferation by 40% in mammary and ovarian carcinoma cell lines. The LASP-1 knock down ovarian carcinoma cells accumulate in the G2 phase of the cell cycle [26]–[28].

We identified LASP-1 as a novel protein that binds to CXCR2 through a proteomic screen for novel CXCR2 binding proteins described earlier [15], [16], [29]. To validate the interaction between CXCR2 and LASP-1, co-immunoprecipitation, GST-pull down and co-localization studies were performed. The binding site for LASP-1 on CXCR2 was mapped using a site-directed mutagenesis approach. LASP-1 was found to directly interact with CXCR2-C terminal domain (CTD) through its LIM domain. To determine the functional significance behind this interaction, we interrupted LASP-1 function by two approaches i) over-expressing a dominant negative plasma membrane targeted LIM domain of LASP-1 and ii) knocking down LASP-1. 293-CXCR2 cells with interrupted LASP-1 function by these two approaches were compromised in their ability to undergo chemotaxis towards CXCL8. Analysis of signal transduction pathways pointed to deficient activation of proteins involved in focal adhesion turn over and cell motility. Overall, this study demonstrated for the first time that the LASP-1 protein is a novel member of the “CXCR2 chemosynapse” playing a critical role in CXCR2 function.

## Materials and Methods

### Cell Culture

#### i) HL-60 CXCR2 cells

Human promyelocytic leukemia (HL-60) cells stably expressing human CXCR2 [30] were grown in RPMI-1640 (Invitrogen, Carlsbad, CA) supplemented with 10% heat-inactivated fetal bovine serum (Atlanta Biologicals, Norcross, GA), 25 mM HEPES, 3 mM L-glutamine and Penicillin (50 units/ml)/Streptomycin (50 µg/ml) (Mediatech, Inc., Herndon, VA). Differentiated HL-60 cells were prepared as previously described [30]. These will be denoted as ‘dHL-60 CXCR2’ cells.

#### ii) 293-CXCR2 and 293-HA-CXCR4 cells

Human embryonic kidney 293 cells (HEK-293) (American Type Culture Collection, Manassas, VA) stably expressing human CXCR2 or HA-tagged CXCR4 were grown in Dulbecco-modified minimum essential medium (DMEM) (Invitrogen, Carlsbad, CA) supplemented with 10% heat-inactivated fetal bovine serum (Atlanta Biologicals, Norcross, GA), 3 mM L-glutamine and Penicillin (50 units/ml)/Streptomycin (50 µg/ml) (Mediatech, Inc., Herndon, VA). These cells will be denoted as ‘293-CXCR2’ or 293-HA-CXCR4 cells.

### DNA constructs and generation of mutants

#### Mammalian expression constructs for LASP-1 and its domains

cDNA constructs for HA-LASP-1, HA-LIM, HA-LIM-NR and HA-NR-SH3 in pcDNA 3.0 vector were kindly provided by Dr. Catherine S. Chew of Medical College of Georgia, Augusta, GA. The constructs for different domains of LASP-1 were engineered for optimal expression by site-directed mutagenesis employing the Quik Change mutagenesis kit (Strategene, La Jolla, CA).

### Engineering of LIM-LIM-CCAX construct

A plasma membrane targeted LIM domain of LASP-1 was engineered in order to employ it as a dominant negative against CXCR2-LASP-1 interaction. Basically, the LIM domain of LASP-1 was fused with the CAAX box of *K-Ras B* so that the expressed LIM domain will be targeted to the plasma membrane in order for it to interrupt the CXCR2-LASP-1 interaction. In addition, it can continue to interfere with CXCR2-LASP-1 interaction even after endocytosis of CXCR2 because of the isoprenylation of LIM domain at its carboxyl terminus will enable it to be associated with the endocytic vesicles. We constructed two dominant negative constructs, one with one LIM domain fused to the CAAX box at the 3′ end and another with two tandem LIM domains with the CAAX box at the 3′ end. This is to ensure that at least one will work as a dominant negative as LIM domain is very small with only 60 amino acid residues and it has to reach the CTD of CXCR2 in order to bind and interrupt the interaction between the endogenous LASP-1 and CXCR2-CTD. Briefly, the CAAX box of *K-Ras B* was amplified from *K-Ras B* template with the following primers: Forward: 5′-GAA AAG ATG AGC AAA GAC GG-3′ and Reverse -5′- AAA GTA GCG GCC GCT TAC ATA ATT ACA CAC TTT G-3′ (PCR product I). The LIM domain with EcoRI restriction site at the 5′ end and at the 3′ end with overlapping sequences to the 5′ end of the CAAX box was amplified from pcDNA3.0-LIM template with the following set of primers: Forward – 5′-TTT GTA GAA TTC ACC ATG GGC TAC CCA TAC G-3′; Reverse- 5′-CTT TGC TCA TCT TTT CCT GCT TGG GGT AGT G-3′ (PCR product II). PCR products I and II (served as templates) were joined to form LIM-CAAX by performing a PCR with primers: Forward -5′-TTT GTA GAA TTC ACC ATG GGC TAC CCA TAC G-3′ and Reverse -5′- AAA GTA GCG GCC GCT TAC ATA ATT ACA CAC TTT G-3′ (PCR product III). The fused LIM-CAAX PCR product (PCR product III) was digested with EcoRI and NotI, gel purified and ligated into similarly digested pcDNA3.0 vector to obtain LIM-CAAX-pcDNA3.0 construct (one LIM domain of LASP-1 fused to CAAX box of *K-Ras B*). The second tandem LIM domain was amplified without a stop codon by performing PCR with pcDNA3.0-LIM template with the following set of primers: Forward – 5′-TTT GTA AAG CTT ACC ATG GGC TAC CCA TAC G-3′; Reverse- 5′-TTT GTA GAA TTC CTG CTT GGG GTA GTG TGC-3′ (PCR product IV). The PCR product IV was restricted with HindIII and ECoRI, gel purified and ligated at the 5′ end of similarly digested LIM-CAAX-pcDNA3.0 construct to obtain the final tandem LIM-LIM-CAAX construct (two LIM domains fused to CAAX box of K-ras B).

### GST-CXCR2-CTD constructs

#### i) Generation of GST-CXCR2 full length WT and mutant CTD constructs

For GST-pull down studies, the gene encoding the full-length carboxyl terminus (CTD) of CXCR2 (amino acid residues 311–355) was engineered with BamHI and XhoI cloning sites by employing the forward – 5′-CTC TAG GGA TCC TTC ATT GGC CAG AAG T-3′ and reverse primers – 5′-CTA GCT CTC GAG TTA GAG AGT AGT GG-3′ and inserted into of the GST bacterial expression vector pGEX-6P-1 (GE Healthcare, Piscataway, NJ). To screen for the binding site for LASP-1 on the CXCR2-CTD, the C-terminus was split into two parts (311–330 and 331–355) and fragments were inserted into BamHI and XhoI sites of the GST vector, pGEX-6P-1. To generate the first half of the CXCR2-CTD, GST-CXCR2-311-330, a stop codon was introduced at position 331, converting a serine (AGC) to a stop codon (TGA) in GST-CXCR2-311–355 with forward and reverse PCR primers 5′-CTA GCT ATA CAT GGC TTG ATC TGA AAG GAC TCC-3′ and 5′-GGG CAG GGA GTC CTT TCA GAT CAA GCC ATG TAT-3′ respectively. Using GST-CXCR2-311-355 WT as the template the following additional mutants were generated: *GST-CXCR2-H318Q/G319A*: Forward primer – 5′-GGC CAG AAG TTT CGC CAA GCA CTC CTC AAG ATT CTA GC-3′; Reverse primer – 5′- GCT AGA ATC TTG AGG AGT GCT TGG CGA AAC TTC TGG CC-3′; *GST-CXCR2-K322R*: The K322R mutant CXCR2 full length plasmid [31] was used as the template to generate this mutant with the PCR primers forward – 5′-CTC TAG GGA TCC TTC ATT GGC CAG AAG T-3′ and reverse – 5′-CTA GCT CTC GAG TTA GAG AGT AGT GG-3′; *GST-CXCR2-A325G/I326V/H327Q*: Forward primer: 5′-CTC CTC AAG ATT CTA GGT GTA CAA GGC TTG ATC AGC AAG G-3′; Reverse primer: 5′- CCT TGC TGA TCA AGC CTT GTA CAC CTA GAA TCT TGA GGA G-3′; *GST-CXCR2-G328A/L329M/I330V*: Forward primer: 5′- GAT TCT AGC TAT ACA TGC CAT GGT CAG CAA GGA CTC CCT GCC-3′; Reverse primer – 5′- GGC AGG GAG TCC TTG CTG ACC ATG GCA TGT ATA GCT AGA ATC-3′. All the mutants described here were cloned into pGEX-6P-1 vector. Most of the constructs were engineered with the ‘QuikChange’ mutagenesis kit (Strategene, La Jolla, CA). The mutants L320A/L321A and I323A/L324A were engineered as previously described [19].

### Generation of GST-CXCR1, GST-CXCR3 and GST-CXCR4 constructs

Engineering of the GST-CXCR1 construct has been described previously [32]. GST-CXCR3-WT CTD: Human CXCR3 cDNA was used as the template to clone out the CXCR3 WT CTD; Forward primer-5′-CTC TAG GGA TCC TTT GTA GGG GTC AAG TTC-3′; Reverse primer-5′-CTA GCT CTC GAG TCA CAA GCC CGA GTA GG-3′. GST-CXCR4-WT CTD: Human CXCR4 cDNA [33] was used the template to PCR out the CXCR4 CTD; Forward primer-5′-CTC TAG GGA TCC TTC CTT GGA GCC AAA TTT AAA AC-3′; Reverse primer-5′-CTA GCT CTC GAG TTA GCT GGA GTG AAA AC-3′.

### GST-fusion constructs Lasp-1 and its domains

Mammalian expression constructs for full length WT LASP-1, LIM, LIM-NR and NR-SH3 domains of the LASP-1 were double digested with Bam HI and Xho I and shuttled to the pGEX-4T-1 vector using same restriction sites.

### Verification of cDNA constructs by sequencing

Fidelity of DNA constructs was verified by DNA sequencing using Big Dye Terminator chemistry at the Vanderbilt DNA sequencing core facility.

### Co-immunoprecipitation of CXCR2 and LASP-1

CXCR2 and endogenous LASP-1 in dHL-60 CXCR2 cells and 293-CXCR2 cells was co-immunoprecipitated and analyzed as described [16]. Co-immunoprecipitation of HA-CXCR4 and endogenous LASP-1 was similarly performed in 293-HA-CXCR4 cells except that the cells were stimulated with 500 ng/ml of CXCL12 for the indicated time points.

### Co-localization of CXCR2, LASP-1 and GFP-PH-AKT in dHL60 cells

The co-localization of CXCR2 with LASP-1 and GFP-PH-AKT in dHL-60 CXCR2 cells was examined by either stimulating the cells with CXCL8 concentrically or by a chemokine gradient generated in the Zigmond chamber (Neuroprobe, Gaithersbug, MD) as described [34]. The paraformaldehyde fixed cover slips were processed for staining as described [16] CXCR2 and LASP-1 were probed with rabbit anti-CXCR2 [29] and mouse monoclonal anti-LASP1 antibodies (clone 8C6). After washing thrice with PBST, the cover slips were incubated with either donkey anti-mouse or donkey anti-rabbit antibodies that were conjugated to the flourophores, cy3 or cy5 (Jackson Immunoresearch Inc., West Grove, PA). GFP-tagged PH domain of Akt was followed by the intrinsic fluorescence of GFP. F-actin was stained with Rhodamine-phalloidin (Invitrogen, Carlsbad, CA). Washed cover slips were mounted with ProLong Gold antifade reagent (Invitrogen, Carlsbad, CA). Confocal images of the cells were acquired using a Zeiss Inverted LSM-510 Meta laser scanning confocal microscope (Carl Zeiss, Thornwood, NY) with a 63X objective and 1.4 Plan-APOCHROMAT oil immersion lens. The images were processed assembled with Photoshop computer program (Adobe Systems, San Jose, CA).

### GST-CXCR2-CTD pull down of LASP-1

After verification of the cDNA by sequencing for the correct coding, the GST-fusion protein constructs were transformed into BL 21 cells for the production of GST-CXCR2-CTD proteins using standard protocols described [16]. Briefly, 100 µg of the GST control protein, GST-CXCR2-311-355 (full length CTD), GST-CXCR2-311-330 (first half of the CTD) and GST-CXCR2-331-355 (second half of the CTD) were washed in the binding buffer and mixed with mammalian cell lysates overexpressing the HA-LASP-1, HA-LIM, HA-LIM-NR and HA-NR-SH3 proteins for 1 h at 4°C. After washing the beads thrice by centrifugation, the bound proteins were eluted and analyzed by 10% SDS-PAGE followed by Western blotting probing for LASP-1 with mouse monoclonal anti-HA antibody (Sigma, St. Louis, MO).

### Direct binding of LASP-1 and LIM domain to GST-CXCR2-CTD WT or its mutants

#### i) Bacterial purification of LASP-1 and LIM domain of LASP-1

BL21 bacteria harboring GST-HA-LASP-1 and GST-HA-LIM domain plasmids were cultured as described in *‘Production of GST proteins section’*. After isolation of the proteins GST-LASP-1 and GST-LIM domain on glutathione beads, the LASP-1 and the LIM domain were cleaved with 50 U of thrombin (GE Healthcare sciences) following manufacturer's protocol. After thrombin removal by incubating with Benzamidine Sepharose, HA-LASP-1 and HA-LIM were separated from the beads by centrifugation and total protein in the supernatant was quantified by Bio-Rad assay. The purified HA-LASP-1 and HA-LIM were stored in aliquots at −80°C.

#### ii) Direct binding of LASP-1 or LIM domain to GST-CXCR2-CTD

5 µg of purified LASP-1 or LIM-domain was incubated with GST control beads or GST-CXCR2 WT CTD immobilized on glutathione beads and nutated for 1 h at 4°C. After separating the unbound from the bound by centrifugation, the beads were washed thrice with the binding buffer and once with 20 mM Tris-buffered saline (TBS). LASP-1 or LIM domain that directly bound to CXCR2-CTD were resolved by 10% or 15% SDS-PAGE and analyzed by Western blot analysis.

### Knockdown of LASP-1 in 293-CXCR2 cells

To determine the functional significance of LASP-1 in the CXCR2 “chemosynapse”, LASP-1 was knocked down in 293-CXCR2 cells by transfecting shRNA mir plasmids for LASP-1 (clone ID – V2LHS_64681, V2LHS_64684, V2LHS_64685 and V2LHS_64686). These short hairpin clones were selected from the GIPZ Lentiviral shRNAmir library (Open Biosystems, Huntsville, AL). A non-silencing construct (NS) in the same vector served as the control. These constructs are bicistronic for GFP and shRNA expression. Briefly, 4 µg of the shRNA mir plasmids pre-mixed with Fugene 6 was transfected into 293-CXCR2 cells. 48 h post transfection, cells were monitored for GFP expression and selected with puromycin that stably expressed the GFP (a marker that was used to track shRNA mir expression). The level of knock down in the polyclonal stable cell line was determined by Western analysis of the stable cell lines and knock down cells with at least 70% knock down were analyzed for CXCR2-mediated chemotaxis, adhesion to collgen IV and CXCR2-mediated signaling pathways.

### Chemotaxis assays in modified Boyden chamber

293-CXCR2 cells over expressing LIM-LIM-CAAX as a dominant negative or with LASP-1 knocked down (LASP-KD5 cells – cells with stable expression shRNA mir plasmid, V2LHS_64685, with more than 90% knock down in LASP-1 level) were analyzed for their ability to undergo CXCL8-mediated chemotaxis as described previously [16], [29], [30], [34], [35]. Chemotactic index was calculated by normalizing the number of cells that migrated under basal conditions to 1.

### Statistical analysis

Data analysis was performed using GraphPad Prism 5 software (GraphPad Software, Inc., San Diego, CA). Statistical significance between groups was determined by analysis of variance (ANOVA) followed by Tukey's multiple comparison test with statistical significance set at p<0.05.

### Image J analysis

In CXCR2-signaling analysis of LIM-LIM-CAAX and LASP-KD5 cells, the Western blots from 3 independent experiments were analyzed using NIH Image J program (ver 1.41o).

## Results

### Validation that CXCR2 associates with LASP-1 by co-immunoprecipitation of CXCR2 and LASP-1

**Figure 1:**
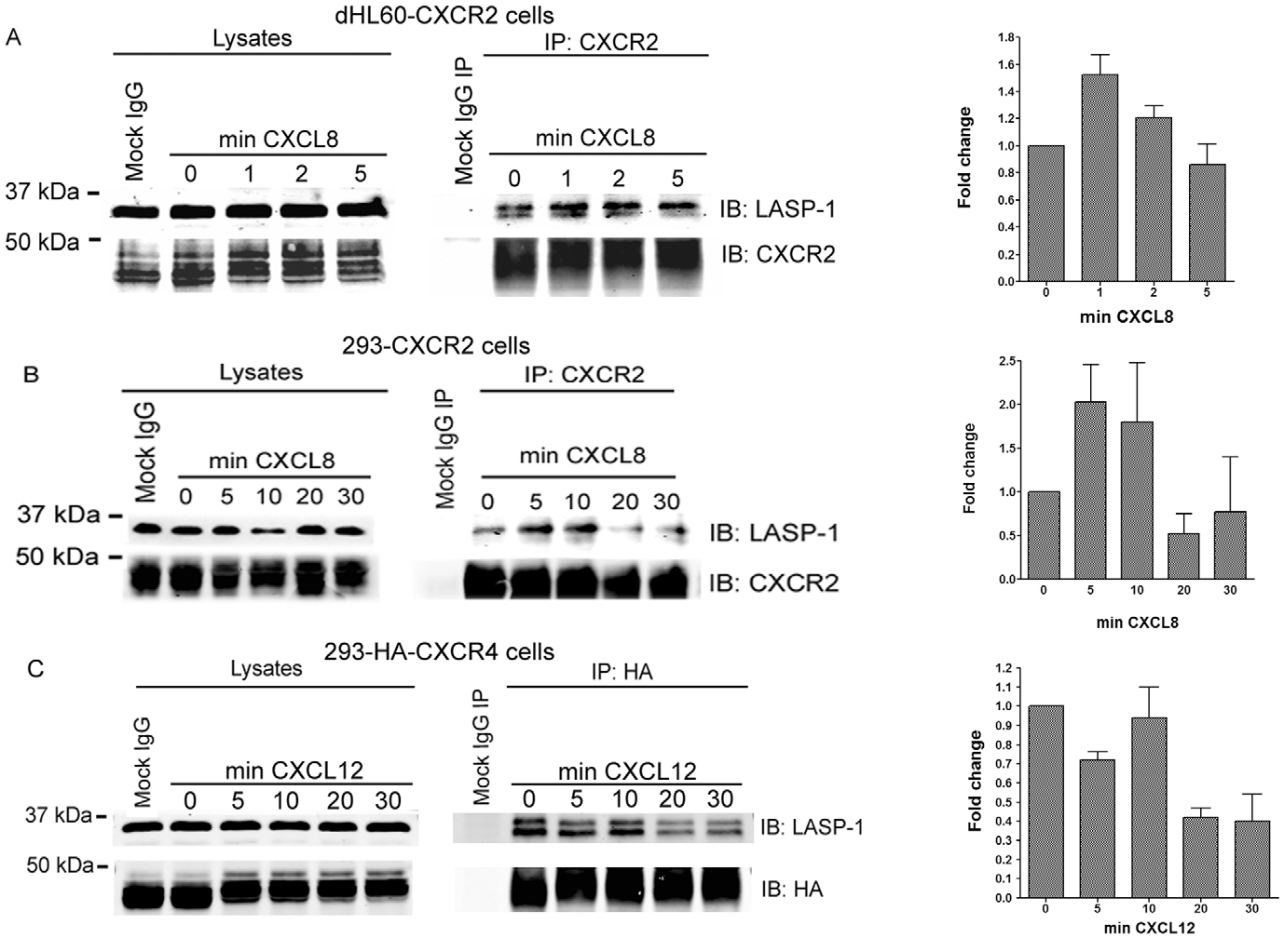
CXCR2 co-immunoprecipitates with LASP-1. A) Lysates from dHL-CXCR2 cells stimulated with vehicle (0 min) or 100 ng/ml of CXCL8 for 1, 2 and 5 min were incubated with control mouse monoclonal IgG (Mock) or with mouse anti-CXCR2 antibody pre-bound to Protein A/G Sepharose. CXCR2-bound proteins were eluted and analyzed for CXCR2 and associated endogenous LASP-1. B) Lysates from 293-CXCR2 cells that were stimulated with vehicle (0 min) or 100 ng/ml of CXCL8 for 5, 10, 20 and 30 min were incubated with control mouse monoclonal IgG (Mock) or with mouse anti-CXCR2 antibody prebound to Protein A/G Sepharose. CXCR2-bound proteins were eluted and analyzed for CXCR2 and associated endogenous LASP-1. C) Lysates from 293-HA-CXCR4 cells that were stimulated with vehicle (0 min) or 100 ng/ml of CXCL12 for 5, 10, 20 and 30 min were incubated with control mouse monoclonal IgG (Mock) or with mouse anti-HA antibody cross-linked to Protein A/G Sepharose. CXCR4-bound proteins were eluted and analyzed for CXCR4 and associated endogenous LASP-1. The bar graph on the right for each cell line represents the co-immunoprecipitation data from three independent experiments with error bars (S.E.M.).

The proteomic screen for CXCR2 interacting proteins has been described earlier [15], [16], [29]. Among many CXCR2-interacting proteins, LASP-1 was identified as a novel binding protein for basal and ligand-activated forms of CXCR2. In order to evaluate the molecular interaction between CXCR2 and LASP-1, a co-immunoprecipitation analysis was performed with and without CXCL8 stimulation of dHL-60 and 293-CXCR2 cells that stably express CXCR2. In dHL-60 CXCR2 and 293-CXCR2 cells, there was basal association of endogenous LASP-1 to CXCR2, as observed previously in proteomic analysis (Figure 1A and 1B). There was an increasing trend in CXCL8-stimulated association of LASP-1 with CXCR2 over time in dHL-60 cells. It appears that the phosphorylated form of LASP-1 has more affinity over time though the human LASP-1 does not show a band shift upon phosphorylation [36]. There is an alternative possibility that the lower minor band may represent the naturally degraded form of LASP-1. Ligand activation of CXCR2 in 293-CXCR2 cells resulted in an increased association of LASP-1 with CXCR2, especially at the 5 and 10 min time points but the association decreased by 20 and 30 min (Figure 1B). The co-immunoprecipitation data indicated that, under basal conditions, a pool of the chemokine receptor CXCR2 was always associated with endogenous LASP-1. Under basal conditions, LASP-1 strongly co-immunoprecipitated with HA-CXCR4 and upon stimulation with CXCL12, the association with LASP-1 mostly remained constant until 10 min, but was reduced by the 20 and 30 min time points. The doublet observed is presumably the phosphorylated form of LASP-1 as the monoclonal anti-LASP-1 antibody (clone 8C6) does not cross-react with endogenous LASP-2 (C. Chew, personal communication) (Figure 1C). This correlated well with the observation that CXCR4 receptor directly associated only with the phosphorylated form of LASP-1 and that the immunoprecipitation concentrated the phosphorylated LASP-1, as it is not detected in the lysates. The co-immunoprecipitation data from a minimum of three independent experiments were quantitated and the fold change in association of LASP-1 with CXCR2 and CXCR4 over time was shown on the corresponding bar graph on the right. We demonstrate for the first time that both CXCR2 and CXCR4 co-immunoprecipitate with endogenous LASP-1.

### CXCR2 co-localizes with LASP-1 in neutrophil-like dHL-60-CXCR2 cells

#### i) Co-localization of LASP-1 and CXCR2 in concentrically stimulated dHL-60-CXCR2 cells

**Figure 2:**
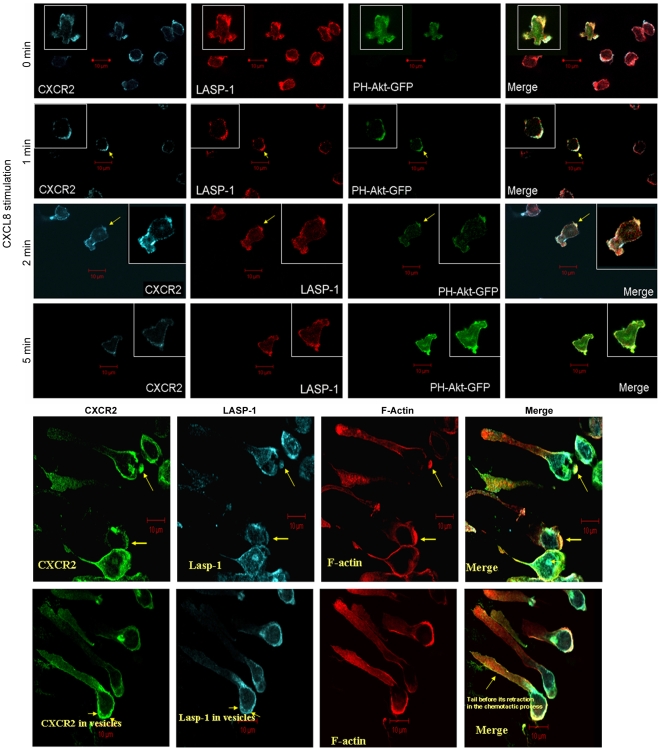
CXCR2 co-localizes with LASP-1 in neutrophil-like dHL-60 CXCR2 cells upon stimulation with CXCL8. A) Confocal images of neutrophil-like dHL-60 CXCR2 cells that stably express GFP-PH-Akt were concentrically stimulated with 100 ng/ml of CXCL8 either with vehicle (0 min) or 100 ng/ml of CXCL8 for 1, 2 and 5 min. CXCR2, LASP-1 and GFP-PH-Akt were pseudo-colored cyan, red and green, respectively. The co-localization of CXCR2 and LASP-1 was shown by the arrows at the leading edge marked by GFP-PH-Akt at 1 and 2 min of stimulation with CXCL8 (yellow arrows). Images represent single z-stack section of 0.5 µm. Inset – A magnified view of selected cells for better clarity. B) Confocal images of dHL-60 CXCR2 cells that were directionally stimulated with 50 ng/ml of CXCL8 in the Zigmond chamber for 10 min. CXCR2, LASP-1 and F-actin were pseudo-colored green, cyan and red respectively. CXCR2, LASP-1 and F-actin clearly co-localize at the leading edge of the polarized cells. In the polarized cell marked by yellow arrows, CXCR2 co-localizes with LASP-1 in internalized vesicles near the leading edge (3 vesicles). Images represent single z-stack sections of 0.5 µm.

Ligand activation of chemokine receptors activates PI3-Kinase, which generates phosphatidylinositol-tris-(3,4,5)phosphate (PIP3) at the leading edge of the cell, an event associated with polarization that dictates directionality of the cell migration [37]–[39]. PIP3 recruit AKT to the plasma membrane in order to get AKT phosphorylated by 3′-phosphoinositide-dependent kinase (PDK). We employed the PH domain of AKT that was tagged to GFP (GFP-PH-AKT) to serve as a marker for polarized PIP3 accumulation upon CXCL8 stimulation in dHL-60 cells. dHL-60-CXCR2 cells transduced to stably express GFP-PH-AKT were stimulated concentrically with CXCL8 and examined for co-localization of CXCR2, GFP-PH-AKT, and endogenous LASP-1. Even though only a small % of the dHL-60 cells expressed GFP-PH-AKT, there was sufficient number of cells for examination of the co-localization with receptor. Cells plated on fibronectin-coated glass cover slips were stimulated concentrically with vehicle or 50 ng/ml of CXCL8 for 0, 1, 2 and 5 min. The fixed cells were stained and evaluated for co-localization of CXCR2 and LASP-1 by confocal microscopy. At 0 min, CXCR2 and LASP-1 were uniformly expressed all around the cell periphery and the cells were mostly rounded, except for a few randomly polarized cells, and GFP-PH-AKT was observed at multiple poles of the cell. At 1 and 2 min post ligand stimulation, both CXCR2 and LASP-1 were co-localized at the polarized edge of the cells marked by the GFP-PH-AKT. After 5 min ligand stimulation, the cells returned to a state where CXCR2, LASP-1 and GFP-PH-AKT were observed evenly distributed at the plasma membrane (Figure 2A). The analysis of the distribution profile of CXCR2, endogenous LASP-1 and PH-Akt-GFP indicated that all three proteins co-localize at the leading edge after 1 and 2 min of CXCL8 stimulation [Figure S3, A, top (1 min) and bottom (2 min)].

#### ii) Co-localization in CXCL8-polarized dHL-60-CXCR2 cells

The CXCL8 treated neutrophil-like dHL-60 CXCR2 cells in the Zigmond chamber were highly polarized (Figure 2B) with cell bodies carrying distinctive tails. In these polarized cells, F-actin was used to mark the leading edge of migrating cells (Figure 2B). CXCR2 and LASP-1 clearly co-localized at the leading edge along with F-actin (top). In addition, CXCR2 was also observed with LASP-1 in vesicles close to the leading edge (bottom) (Figure 2B). The analysis of the distribution profile of CXCR2, endogenous LASP-1 and F-actin indicated that all three proteins co-localize at the leading edge of CXCL8 polarized dHL-60-CXCR2 cells. Interestingly, line scan analysis of the distribution of CXCR2 and endogenous LASP-1 mirror each other in the area marked by F-actin at the leading edge (Figure S3, B).

### CXCR2-CTD binds to HA-LASP-1

**Figure 3:**
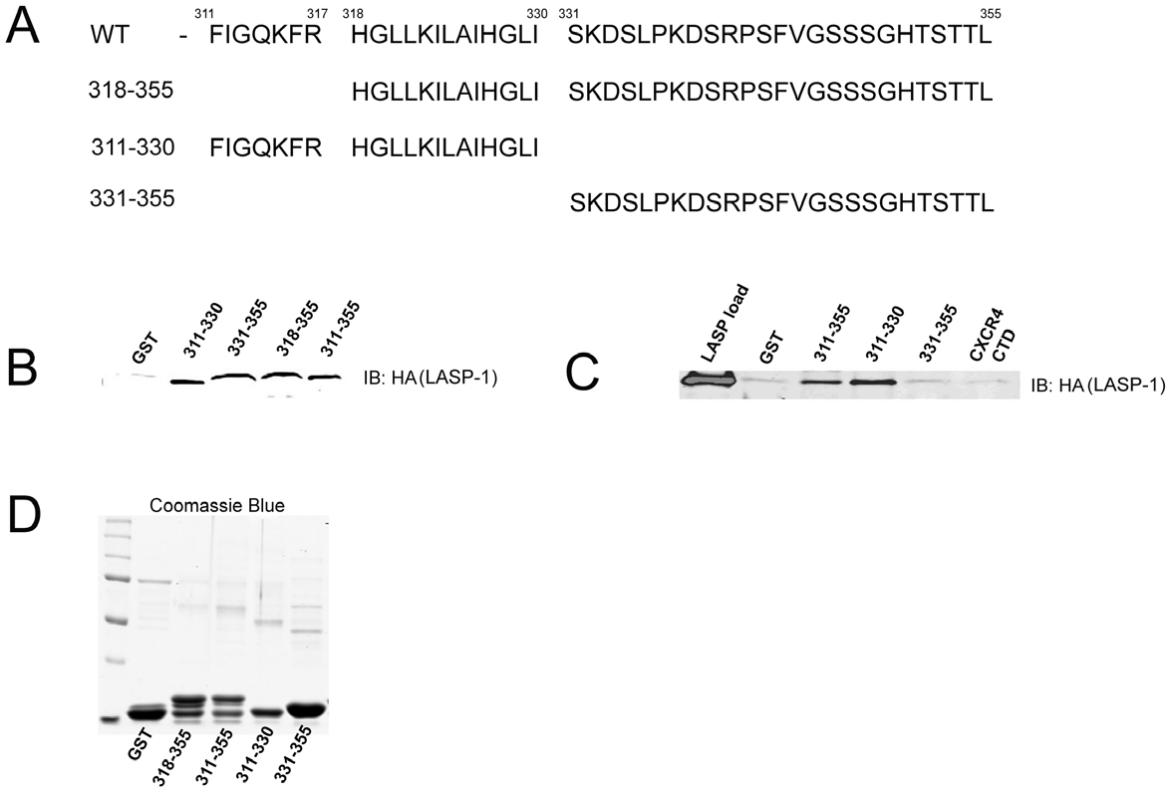
CXCR2-carboxyl terminal domain (CTD) binds to LASP-1. A) *Primary structure of GST-fusion constructs of WT CXCR2-CTD full length (311–355) and its various truncation mutants*. B) *Both halves of CXCR2-CTD bind to HA-LASP-1 over expressed in mammalian cells*. GST pull down assay was performed by incubating GST beads only, GST-CXCR2 (311–330), GST-CXCR2 (331–355), GST-CXCR2 (318–355) and GST-CXCR2 (311–355) with 293-parental cell lysates (1 mg total protein) containing transiently over expressed HA-LASP-1. Lane 1 - GST; Lane 2 - GST-311-330 (first half); Lane 3 – GST-331-355 (second half); Lane 4 – GST-318-355; Lane 5 – GST-311-355 (full length). C) *The first half of CXCR2-CTD (residues 311–330) directly binds to recombinant HA-LASP-1*. GST pull down assay was performed by incubating GST-tagged full length CXCR2-CTD (311–355), the first half of the CXCR2-CTD (311–330) or the second half of the CXCR2-CTD (331–355) with recombinant purified HA-tagged LASP-1 as described in ‘Methods’. D) *Coomassie blue stained gel showing 20 µg of purified GST and different GST-CXCR2-CTD fusion proteins employed in the GST pull down assay*. Lane 1 - GST; Lane 2 – GST-318-355; Lane 3 – GST-311-355 (full length); Lane 4 - GST-311-330 (first half); Lane 5 – GST-331-355 (second half).

HA-tagged LASP-1 was expressed in the parental HEK-293 cells lacking endogenous CXCR2 and these cell lysates were incubated with bacterially expressed and purified GST-tagged CXCR2 WT full length CTD (residues 311–355) and different truncation mutants of CXCR2-CTD. The full length and mutant CXCR2-CTD constructs are schematically presented in Figure 3A. The GST pull down assay revealed that full length CXCR2-CTD has the ability to bind to HA-LASP1. A deletion mutant of CXCR2-CTD that lacked residues 311–317 at the N-terminus of the CXCR2-CTD retained its ability to bind to LASP-1. When the CXCR2-CTD was split in two, the first half (residues 311–330) and the second half (residues 331–355) of the CXCR2-CTD retained the capability to bind to LASP-1 equally well (Figure 3B). 20 µg of GST and GST-fusion proteins of CXCR2-CTD were electrophoresed and stained by Coomassie (Figure 3D). These data indicate that both halves of the CXCR2-CTD may directly or indirectly bind to HA-tagged LASP-1.

### CXCR2-CTD directly binds to recombinant LASP-1

In order to determine whether LASP-1 directly binds specific regions of the CXCR2-CTD, LASP-1 was bacterially expressed fused to GST, purified and cleaved with thrombin to obtain LASP-1 free of GST. Purification analysis of the recombinant LASP-1 by Coomassie staining revealed that about 70% of pure LASP-1 was intact and thrombin did cleave some LASP-1 during its overnight incubation with thrombin. The cleavage products of LASP-1 neither bound CXCR2-CTD nor interfered with binding of full length LASP-1 to CXCR2-CTD (data not shown). The direct binding assay indicated that full length CXCR2-CTD (residues 311–355) and the first half of the CXCR2-CTD (311–330) fused to GST had the ability to bind to LASP-1, but surprisingly the second half of the CXCR2-CTD (331–355) was devoid of any direct binding activity (Figure 3C). Interestingly, the second half of the CXCR2-CTD (331–355) can indirectly bind to CXCR2-CTD as observed when it was expressed in the mammalian cells (Figure 3B).

### Related CXC receptors CTDs bind to LASP-1

**Figure 4:**
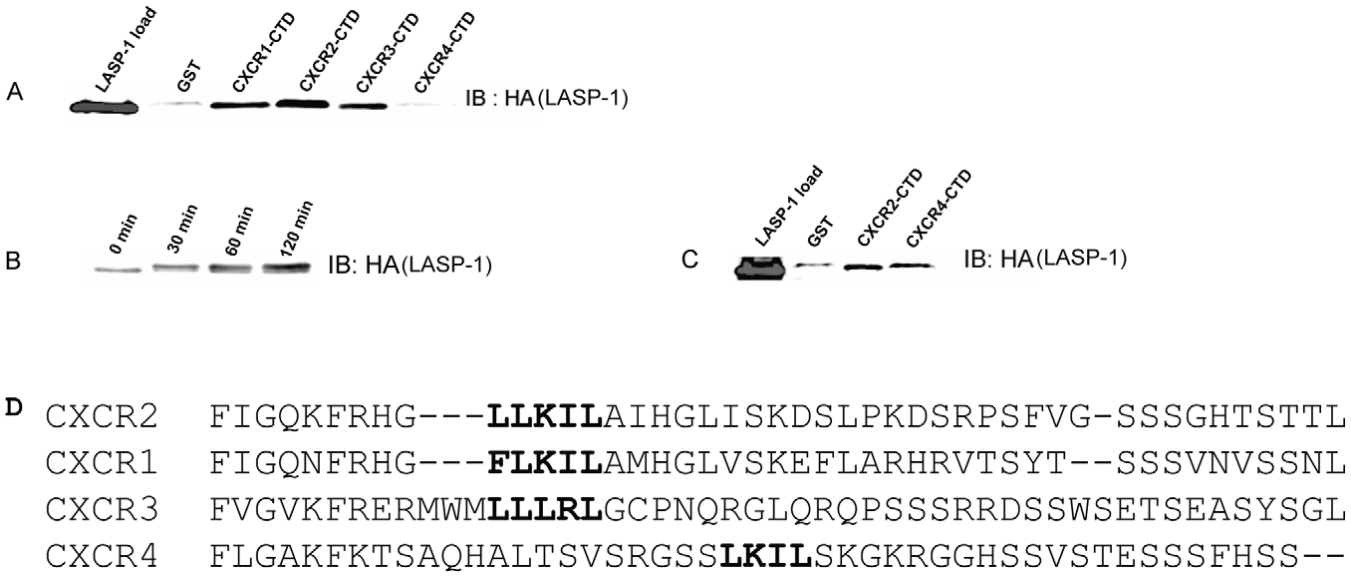
CXC chemokine receptor C-termini other than CXCR2 also directly binds to recombinant LASP-1. A) GST fusion proteins of full length WT CTDs of CXCR1, CXCR2, CXCR3 and CXCR4 were incubated with bacterially expressed and purified recombinant HA-LASP-1 and GST pull down assay was performed. Blots were probed for LASP-1 with mouse monoclonal anti-HA antibody. Lane 1 – HA-LASP-1 load; Lane 2 – GST; Lane 3 – GST-CXCR1; Lane 4 – GST-CXCR2; Lane 5 – GST-CXCR3; Lane 6 – GST-CXCR4. B) *In vitro phosphorylation of recombinant HA-LASP-1*. Recombinant HA-LASP-1 was incubated with the catalytic subunit of protein kinase A (cPKA) in PKA reaction buffer with 2 mM ATP for 2 h at 37°C in order to maximally phosphorylate LASP-1. Lane 1 – Unphosphorylated HA-LASP-1; Lane 2 – HA-LASP-1+ PKA for 30 min; Lane 3 - HA-LASP-1+ PKA for 1 h; Lane 4 - HA-LASP-1+ PKA for 2 h. C) *CXCR4 WT CTD directly binds to phosphorylated LASP-1*. Direct GST pull down assay was performed with PKA phosphorylated recombinant HA-LASP-1 by incubating with GST, GST-CXCR2-CTD (WT) and GST-CXCR4-CTD (WT). D) *CLUSTALW multiple sequence alignment and analysis of CTDs of CXC chemokine receptors*. The CTDs of CXCR2 (45 residues), CXCR1 (44 residues), CXCR3 (49 residues) and CXCR4 (47 residues) were aligned with an on-line version of CLUSTALW (1.83) program. The LLKIL motif of CXCR2 and similar motifs in other receptor CTDs were highlighted.

CLUSTAL W (1.83) multiple sequence alignment and analysis of the sequences between CXCR1, CXCR2, CXCR3 and CXCR4 CTDs indicated that many residues are conserved among them. The LLKIL motif in the CTD of these chemokine receptors are highlighted (Figure 4D). In the CXCR4-CTD there is an extra serine cluster separating the LKIL motif. In order to ascertain whether CXCR2 binding to LASP-1 is specific, or whether other related CXC chemokine receptor CTDs have the capacity to bind LASP-1, GST-CXCR1, CXCR3 and CXCR4-CTDs were engineered. The binding analysis revealed that in addition to CXCR2-CTD, CXCR1, CXCR3-CTDs bound LASP-1 directly, but the CXCR4-CTD failed to bind LASP-1 (Figure 4A). LASP-1 is known to be phosphorylated by protein kinase A (PKA) [36], . To determine whether CXCR4-CTD binding to LASP-1 may require that LASP-1 be phosphorylated, bacterially expressed and purified LASP-1 was phosphorylated *in vitro* by the purified recombinant catalytic subunit of PKA. With increasing incubation time with cPKA there is a corresponding increase in phosphorylation of recombinant LASP-1 as observed by a band shift (Figure 4B). Direct binding of CXCR4 CTD to *in vitro* phosphorylated recombinant LASP-1 confirmed that CXCR4 CTD will directly bind phospho-LASP1 (Figure 4C).

### CXCR2-CTD binds to LASP-1 through its LIM domain in mammalian cells

**Figure 5:**
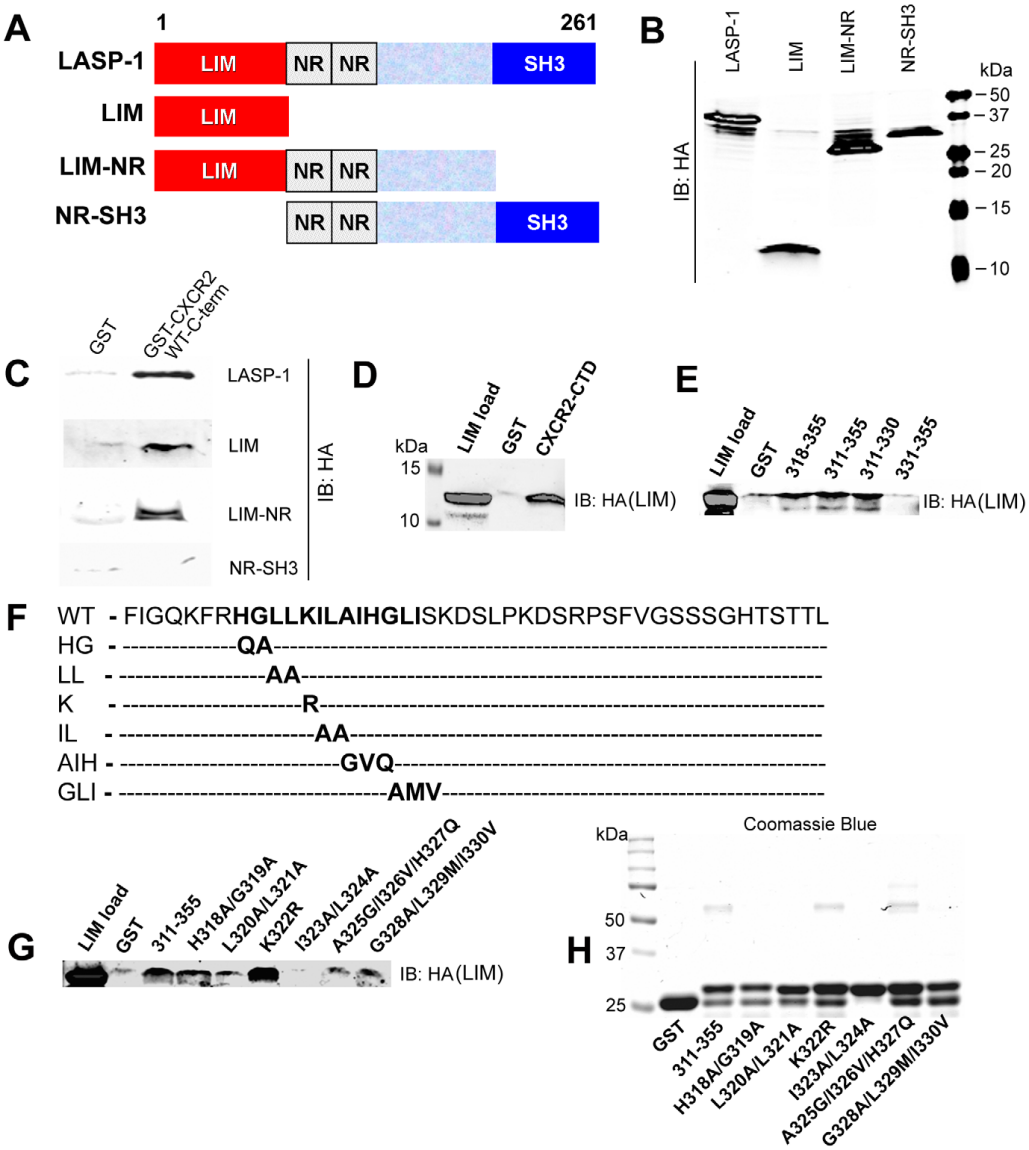
CXCR2 WT-CTD binds to the LIM domain of LASP-1. A) *Schematic representation of the mammalian expression constructs for full length LASP-1 and its various domains*. LIM – *Lin/Isl/Mec*; NR – Nebulin repeats; SH3 – *Src* Homology 3 domain. B) *Expression profile of full length LASP-1 and its various domains in 293-parental cells*. Full length LASP-1, LIM, LIM-NR and NR-SH3 domains of LASP-1 (all were HA-tagged) were transiently over-expressed in 293-parental cells. 50 µg of total lysates were resolved by 10% SDS-PAGE, transferred and blotted with monoclonal mouse anti-HA antibody. C) *LIM domain of LASP-1 mediates its binding to CXCR2-WT-CTD*.GST-pull down assay with GST alone or GST-CXCR2 WT-CTD incubated with lysates (1 mg total protein) from 293-parental cells expressing full length LASP-1, LIM, LIM-NR and NR-SH3 domains. D) *Direct binding of purified CXCR2-CTD to LIM domain of LASP-1*. Purified HA tagged LIM domain of LASP-1 obtained after thrombin cleavage from the bacterially expressed and purified GST-LIM fusion protein was incubated with GST or GST-CXCR2-CTD (311–355) (WT) and a pull down assay was performed. E) *Direct binding of CXCR2-CTD to LIM domain of LASP-1 occurs through the first half of the CXCR2-CTD (residues 311–330)*. GST pull down assay was performed with GST control and GST fusion of full length CXCR2-CTD (311–355) and various truncation mutants of CTD. Lane 1 – Purified LIM load; Lane 2 – GST; Lane 3 – GST-CXCR2-CTD-318-355; Lane 4 - GST-CXCR2-CTD-311-355; Lane 5 - GST-CXCR2-CTD-311-330; Lane 6 - GST-CXCR2-CTD-331-355. F) *Site-directed mutants of the CXCR2-CTD that were engineered to identify the binding site for the LIM domain of LASP-1*. G) *Iso323-Leu324 is the primary binding site for the LIM domain of LASP-1*. A series of GST-CXCR2 site-directed mutants covering the residues 318–330 were mixed with purified recombinant HA-tagged LIM domain of LASP-1 and a pull down assay was performed. Lane 1 – Purified LIM load; Lane 2 – GST; Lane 3 – GST-CXCR2-CTD-311-355 (WT); Lane 4 - GST-CXCR2-CTD-H318A/G319A; Lane 5 - GST-CXCR2-CTD-L310A/L321A; Lane 6 - GST-CXCR2-CTD-K322R; Lane 7 - GST-CXCR2-CTD-I323A/L324A; Lane 8 - GST-CXCR2-CTD-A325G/I326V/H327Q; Lane 9 - GST-CXCR2-CTD-G328A/L329M/I330V. H) *Coomassie blue stained gel of GST and different GST-CXCR2-CTD site-directed mutant fusion proteins spanning the residues 318-330 (20 µg each)*. Lane 1 – GST; Lane 2 – GST-CXCR2-CTD-311-355 (WT); Lane 3 - GST-CXCR2-CTD-H318A/G319A; Lane 4 - GST-CXCR2-CTD-L310A/L321A; Lane 5 - GST-CXCR2-CTD-K322R; Lane 6 - GST-CXCR2-CTD-I323A/L324A; Lane 7 - GST-CXCR2-CTD-A325G/I326V/H327Q; Lane 8 - GST-CXCR2-CTD-G328A/L329M/I330V.

LASP-1 has LIM domain at its N-terminus, flanked by two NR domains and a SH3 domain at its C-terminus (Figure 5A). In order to determine the specific regions on LASP-1 that mediate its binding to CXCR2-CTD, different domains of LASP-1 were expressed as LIM, LIM-NR, or NR-SH3 domain in 293-parental cells. These mutants are schematically presented in Figure 5A. The Western blot of these different domains of LASP-1 is shown in Figure 5B. Both LIM and LIM-NR domains bind to CXCR2-CTD (residues 311–355) fused to GST, but not NR-SH3. As NR-SH3 was devoid of any CXCR2-CTD binding capability, data show that the LIM domain is the domain through which LASP-1 binds to the CXCR2-CTD (Figure 5C).

### CXCR2-CTD directly binds to LIM domain of LASP-1

GST-CXCR2-CTD directly binds to the LIM domain of LASP-1 (Figure 5D). The direct binding of the LIM domain of LASP-1 to CXCR2-CTD was further confirmed by performing a binding assay with first half (311–330) and second half of the CXCR2-CTD (331–355). The LIM domain of LASP-1 bound directly to full length CXCR2-CTD (311–355) and first half of the CXCR2-CTD (311–330), but failed to bind to the second half of the CXCR2-CTD (331–355) (Figure 5E & 3C). Thus, the direct binding of the LIM domain mirrored the direct binding of LASP-1 to the CXCR2-CTD and its truncated mutants and thus verified that the LIM domain of LASP-1 is indeed mediating the interaction between LASP-1 and CXCR2-CTD.

### Isoleucine323 and Leucine324 of the CXCR2-CTD mediates the binding to the LIM domain of LASP-1

From the direct binding studies between CXCR2-CTD and the LIM domain of LASP-1, it became clear that the first 7 residues of the CXCR2-CTD (311–317) were not necessary (Figure 5E) for binding to the LIM domain. The residues 318–330 were important for the binding of LASP-1 and its LIM domain. In order to know the binding site on the CXCR2-CTD residues 318–330 for the LIM domain of LASP-1, a series of site-directed mutants were generated for residues 318–330 in the full length CXCR2-CTD (Figure 5F) and tested for their ability to directly bind to the LIM domain of LASP-1. H318Q/G319A, L320A/L321A, A325G/I326V/H327Q and G328A/L329M/I330V mutants bound to the LIM domain of LASP-1 moderately well (Figure 5G). The K322R mutant exhibited a large increase in the binding, while the I323A/L324A mutant lost LIM binding capacity. This indicates that the I323-L324 residues are very important for binding and the surrounding residues, for example K322, may regulate the binding since mutation of this residue increased the binding (Figure 5G). Figure 5H shows the GST loading control for each mutant (Coomassie) (Figure 5H).

### LIM domain of LASP-1 as a dominant negative

**Figure 6:**
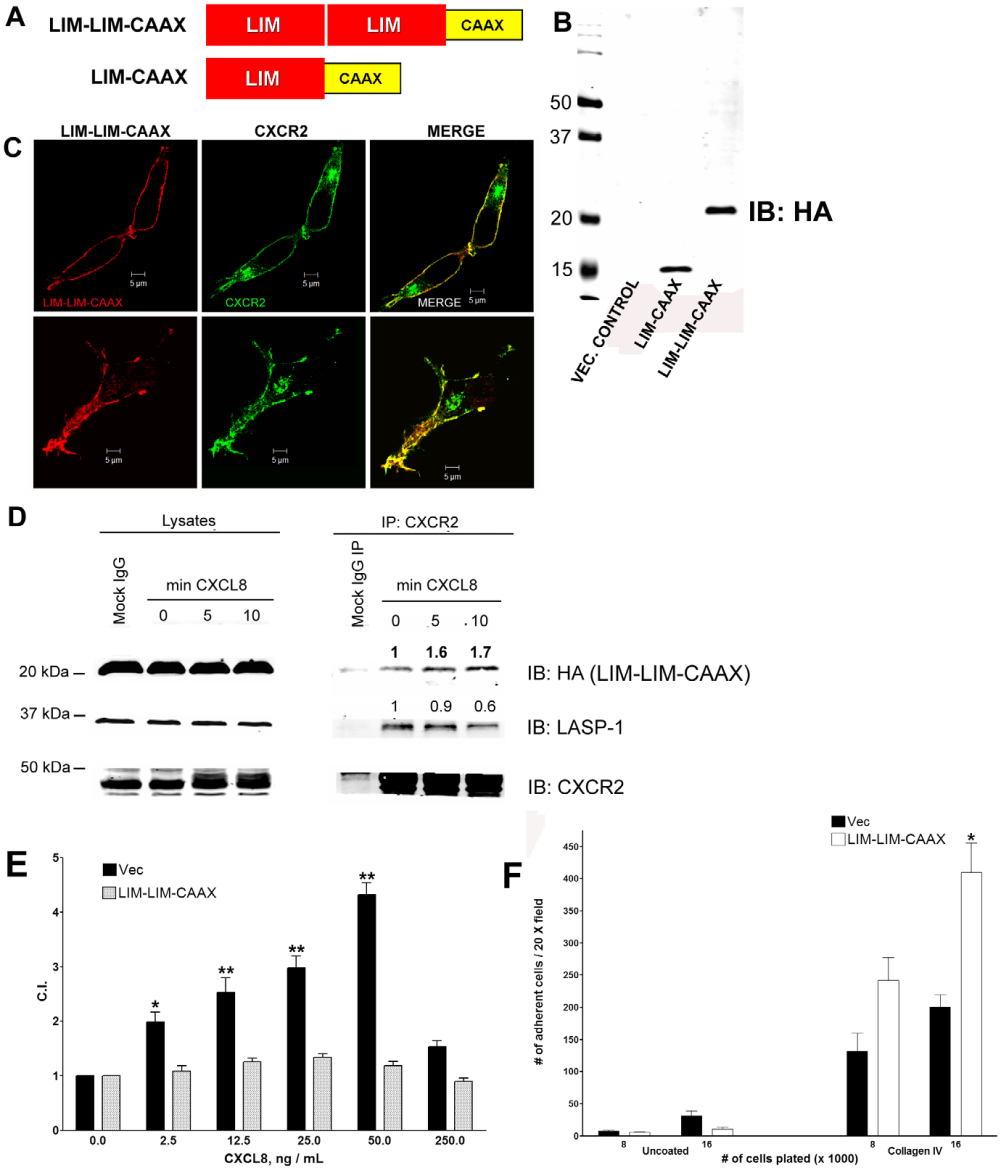
Expression of LIM-LIM-CAAX protein inhibits CXCR2 mediated chemotaxis. A) Schematic representation of the plasma membrane targeted LIM-CAAX and LIM-LIM CAAX constructs used as a dominant negative for inhibiting CXCR2 function. B) *Expression profile of LIM-CAAX and LIM-LIM-CAAX constructs in 293-CXCR2 cells*. HA-tagged LIM-CAAX and LIM-LIM-CAAX plasmids were transiently over-expressed in 293-CXCR2 cells. 50 µg of total lysates from these cells were resolved by 10% SDS-PAGE, transferred to nitrocellulose and blotted for HA (LIM-LIM-CAAX). C) *CXCR2 co-localizes with LIM-LIM-CAAX at the plasma membrane and in internal vesicular structures*. HA-tagged LIM-LIM-CAAX was transiently over-expressed in 293-CXCR2 cells. Confocal images of LIM-LIM-CAAX cells stained for CXCR2 receptor and plasma membrane targeted LIM-LIM-CAAX protein. Single z-stack images (0.5 µm) of two different fields were shown here. D) CXCR2 co-immunoprecipitates with LIM-LIM-CAAX. HA-tagged LIM-LIM-CAAX plasmid was transiently over expressed in 293-CXCR2 cells. Lysates (1.5 mg) from 293-CXCR2 cells that were stimulated with vehicle (0 min) or 100 ng/ml of CXCL8 for 5 and 10 min were incubated with control mouse monoclonal IgG (Mock) or with mouse anti-CXCR2 antibody pre-bound to Protein A/G Sepharose. CXCR2-bound proteins were eluted and separated by 10% SDS-PAGE, blotted for CXCR2 and associated LIM-LIM-CAAX. Fold change over basal (1) of LIM-LIM-CAAX that co-immunoprecipitated with CXCR2 is given above the bands. Similarly, the fold inhibition of the association of endogenous LASP-1 with CXCR2 when LIM-LIM-CAAX was over expressed is given. E) Dominant negative LIM-LIM-CAAX impairs CXCR2 mediated chemotaxis. 293-CXCR2 cells with transient over expression of LIM-LIM-CAAX were tested for their ability to migrate in response to various concentrations of CXCL8 by employing an indirect Boyden chamber assay. The results were analyzed by one-way ANOVA and if the ‘p’ value was <0.05, it was followed by Tukey's multiple comparison test for levels of significance; * - p<0.01; ** - p<0.001. C.I. – Chemotactic Index F) 293-CXCR2 cells expressing LIM-LIM-CAAX show increased adhesion to collagen IV matrix. Vector control cells and LIM-LIM-CAAX cells were seeded in triplicates at different densities (8000 and 16,000 cells) onto uncoated, collagen IV and poly-L-lysine coated wells in a 96-well plate. After washing, the attached cells were counted using a fluorescent inverted microscope.

The Iso323-Leu324 residues of the CXCR2 CTD required for the CXCR2/LASP-1 binding are critical for binding of adaptor protein-2 (AP-2) and Hsp70 interacting protein (HIP) and CXCR2 mediated chemotaxis [18], [19], [30]. So I323A/L324A-CXCR2 full length receptor could not be used to evaluate the role of LASP-1 in CXCR2 mediated chemotaxis. To characterize the functional contribution of LASP-1 in CXCR2 mediated chemotaxis we over expressed the LIM domain of LASP-1 to ablate the interaction between LASP-1 and CXCR2-CTD. Since the LIM domain is highly conserved between both LASP-1 and LASP-2, this should effectively compete with CXCR2 binding to either LASP-1 or LASP-2 [42]. The LIM domain construct was engineered with one or two tandem LIM domains with the CAAX box from the *K-Ras B* protein at its C-terminus (LIM-LIM-CAAX) to ensure plasma membrane localization of the LIM domain construct (Figure 6A). The expression profile of a HA-tagged single LIM domain (lane 2) and two tandem LIM domains fused to the CAAX box (lane 3) is shown (Figure 6B). The construct with a single LIM domain fused to CAAX (LIM-CAAX) was not effective as a dominant negative when tested in the preliminary experiments and was not further used (data not shown). The colocalization analysis by the confocal microscopy revealed that the LIM-LIM-CAAX protein co-localized with CXCR2 at the plasma membrane and during CXCR2 intracellular trafficking (Figure 6C). The LIM-LIM-CAAX protein co-immunoprecipitated with CXCR2 and also inhibited the CXCL8 stimulated association of endogenous LASP-1 until 10 min (Figure 6D). Over expressed LIM-LIM-CAAX in 293-CXCR2 cells impaired CXCR2 mediated chemotaxis significantly (Figure 6E). Moreover, the basal adhesion of LIM-LIM-CAAX cells to collagen IV matrix was increased two-fold compared to the vector transfected cells (Figure 6F).

### Knock down of LASP-1

**Figure 7:**
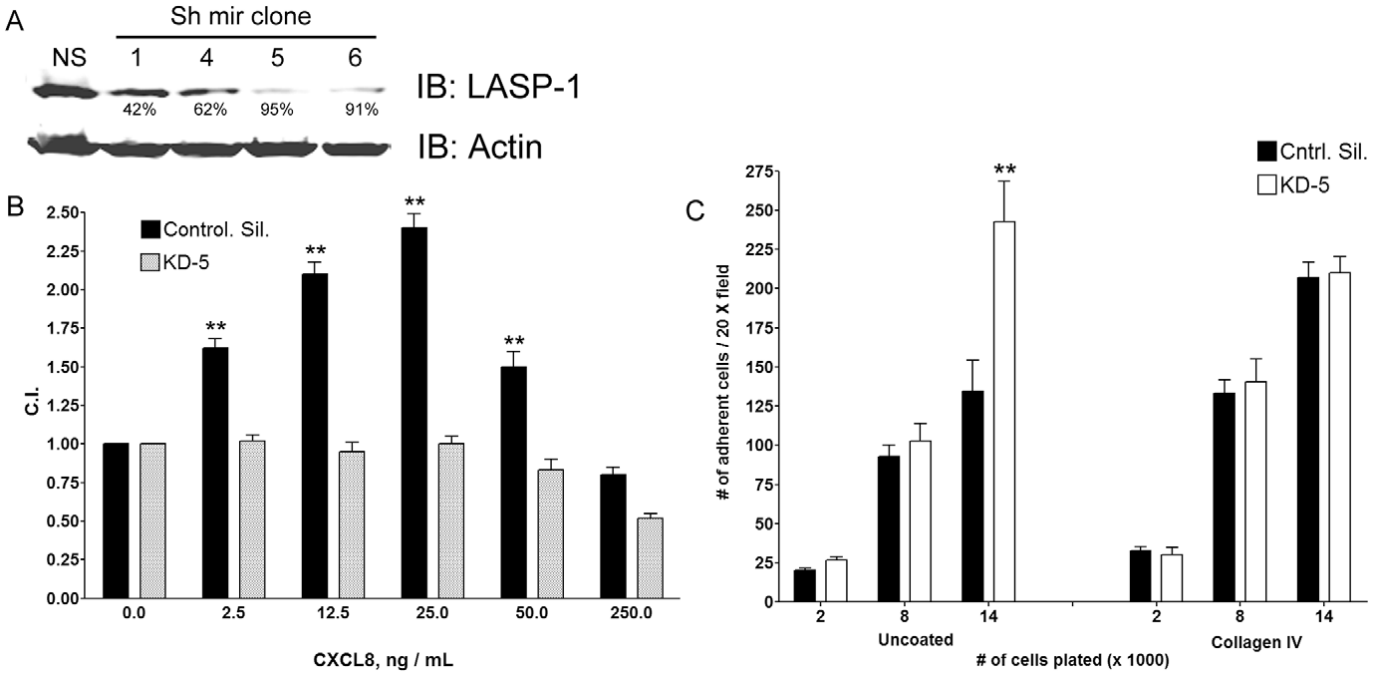
Stable knock down of LASP-1 impairs chemotaxis in 293-CXCR2 cells. A) *Stable knock down of LASP-1 in 293-CXCR2 cells*. Four Sh RNA clones (#64681, #64684, #64685 and #64686) were employed to achieve LASP-1 knock down in 293-CXCR2 cells. Sh RNA plasmids were transfected into 293-CXCR2 cells and 48 h post transfection, cells were selected with puromycin for the stable knock down of LASP-1. The % knock down of LASP-1 was analyzed after 4 weeks and was monitored throughout the analysis period and values for % knock down were given below the bands. NS – Non-silencing sh mir; 1 – Sh mir clone #64681; 4 - Sh mir clone #64684; 5 - Sh mir clone #64685; 6 - Sh mir clone #64686. B) *Knock down of LASP-1 impairs CXCR2-mediated chemotaxis in 293-CXCR2 cells*. 293-CXCR2 cells with stable knock down of LASP-1 were tested for their ability to migrate in response to various concentrations of CXCL8 by employing an indirect Boyden chamber assay. The results were analyzed by one-way ANOVA and if the ‘p’ value is <0.05, it is followed by Tukey's multiple comparison test for levels of statistical significance; ** - p<0.001. C.I. – Chemotactic Index C) *LASP-1 knock down did not alter the adhesion of 293-CXCR2 cells to collagen IV matrix*. Non-silencing control cells and LASP-1 knock down cells were seeded in triplicate at different densities (2000, 8000 and 16,000 cells) onto uncoated and collagen IV coated wells in a 96-well plate. After washing, the attached cells were counted using a fluorescent inverted microscope.

The functional significance behind the interaction between CXCR2-CTD and the LASP-1 was also assessed by stably knocking down LASP-1 by expression of short hairpin RNAs in 293-CXCR2 cells. Of the four Sh mir clones, V2LHS_64681 (labeled as 1), V2LHS_64684 (labeled as 4), V2LHS_64685 (labeled as 5) and V2LHS_64686 (labeled as 6), two (V2LHS_64685 and V2LHS_64686) showed more than 90% of the level of LASP-1 knocked down compared to the silencing control (Figure 7A). 293-CXCR2 cells expressing V2LHS_64685 Sh mir clone was named as ‘LASP-KD5’ cells (polyclonal). LASP-KD5 cells displayed impaired chemotaxis when tested for their ability to migrate in the modified Boyden chamber chemotaxis assay (Figure 7B). In contrast, the adhesivity of the LASP KD-5 cells to collagen IV matrix was not altered compared to the non-silencing control (Figure 7C).

### Analysis of signaling pathways with over expression of LIM-LIM-CAAX as a dominant negative and knock down of LASP-1

Translocation of LASP-1 from the cell periphery to the vinculin-positive focal adhesions has been observed earlier in motile cells but not in nonmotile cells [24]. Based on this observation, we examined activation of proteins in the CXCR2 signaling pathway and proteins residing at the focal adhesions when LIM-LIM-CAAX was employed as a dominant negative and during knock down of LASP-1.

### *c-Src and paxillin activation*

**Figure 8:**
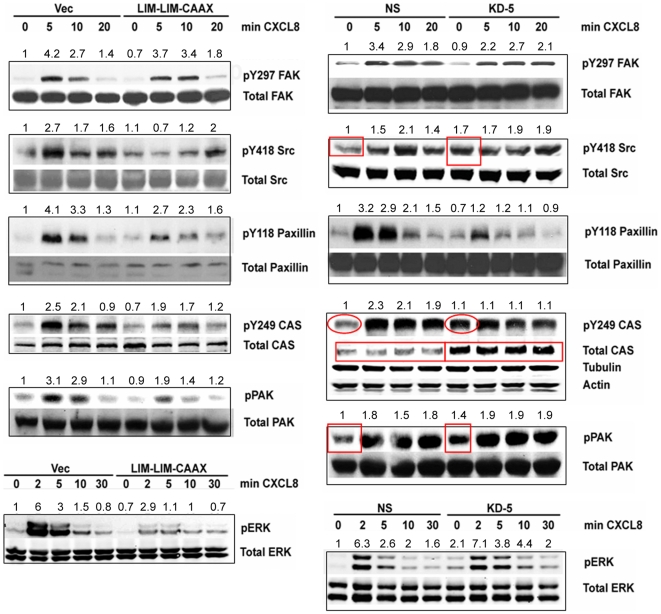
Analysis of signal transduction pathways in LIM-LIM-CAAX and LASP-KD5 cells. Left panel - LIM-LIM-CAAX; Right panel - LASP-KD5 cells. 50 µg of total protein lysates from 293-CXCR2 cells that were stimulated with 50 ng/ml of CXCL8 was separated by 10% SDS-PAGE, transferred to nitrocellulose and blotted with a panel of antibodies: pY397-FAK, pY418-*Src*, pY118-paxillin, pY249-p130CAS, pSer144-PAK1/pThr423-PAK1 and pERK1/2. In panel B, as total p130CAS level was elevated in LASP-KD5 cells, additional loading controls tubulin and actin were included and shown below pY249-p130CAS and total p130CAS lanes. The representative blots for each was shown and the CXCL8 mediated response from 3 independent experiments was quantitated using Image J program and shown.

CXCL8 stimulated c-*Src* activation was compromised in LIM-LIM-CAAX expressing cells (Figure 8, left panel). However, in LASP-KD cells, there was a basal increase in pY418 level in c-*Src*, but the ligand induced increase in the phosphorylation level was not observed (Figure 8, right panel)

### Deficient paxillin phosphorylation at Y118 and p130 CAS phosphorylation at Y249

Paxillin phosphorylation at Y118 and Y31 by FAK and *c-*Src promotes paxillin disassembly from nascent adhesions at the leading edge thus promoting cell migration [12]. LIM-LIM-CAAX cells had deficient phosphorylation of paxillin at Y118, especially at 5 min time point, when compared to the vector control (Figure 8, left panel). Similar deficient activation of paxillin was also observed in LASP KD-5 cells. p130 CAS (Crk-associated substrate) plays a crucial role in cell migration [43], [44] and is a downstream effector of the CXCR2 signaling pathway [45]. It acts as a molecular switch by binding to Crk upon Y249 phosphorylation. This CAS-Crk complex then recruits Dock180 leading to activation of Rac1. In LIM-LIM-CAAX expressing cells, phosphorylation of p130 CAS at Y249 was decreased when compared to the control cells (Figure 8, left panel). In contrast, in the LASP-KD5 cells, there is an apparent increase in basal activation of p130 CAS as observed by an increase in pY249 at 0 min when compared to the non-silencing (ns) control. Surprisingly, the total level of p130 CAS was also increased, upon quantitation and normalization to total CAS there was no net increase of Y249 phosphorylation in CAS when LASP-1 is knocked down.

### Activation of p21-activated kinase 1 (PAK1) and extracellular signal-regulated kinase 1/2 (ERK1/2)

PAK1 activation is very crucial in cell migration and is a downstream effector of CXCR2 signaling pathway [35]. In LIM-LIM-CAAX expressing cells, PAK1 activation was significantly reduced when compared to the vector control (Figure 8). However, there was a basal increase of PAK1 phosphorylation in LASP-KD5 cells. ERK activation has been implicated in phosphorylation of myosin light chain kinase (MLCK), actin-myosin assembly, and contractility during cell migration [12], [46]. Active ERK has been shown to localize to adhesions where myosin light chain kinase (MLCK) can be phosphorylated resulting in increased contractility. Activation of ERK1/2 was significantly blocked by LIM-LIM-CAAX over expression in 293-CXCR2 cells. In contrast, in LASP-KD5 cells, there was a basal PAK activation and as a result there was a diminished CXCL8 stimulated PAK1 phosphorylation. ERK1/2 phosphorylation was marginally increased (Figure 8).

### Rap1 activation, β1-integrin level and FAK activation was not significantly affected

The cross-talk between CXCR2 and β1-integrin through Rap1 and FAK activation was examined. Interference with CXCR2/LASP-1 by the expression of LIM-LIM-CAAX or knock down of LASP-1 did not significantly affect Rap1 activation (though there was a differential activation of Rap1) (Figure S1 – A & B), β1 integrin level (Figure S1 – C & D) and FAK activation (Figure 8). In addition, in LASP-KD5 cells the levels of zyxin, lipoma preferred partner (LPP) or N-Cadherin remained unchanged (Figure S2).

## Discussion

LASP-1 is an actin binding cytoskeletal protein that is known to be involved in cell migration and the targeted disruption of LASP-1 enhanced the migration of mouse embryonic fibroblasts [47]. LASP-1 knock out mice exhibit enhanced wound healing and rapid tumor initiation, consistent with the idea that LASP-1 modulates cell migration [47].

We demonstrate here that LASP-1 is a novel protein that binds CXCR2 in both basal and ligand activated conditions and participates in the “CXCR2 chemosynapse”. CXCR2 has been shown to form a dynamic and functional “chemosynapse” with cytosolic proteins to facilitate chemotaxis [15]. Vasodilator-stimulated phosphoprotein (VASP) is one component of the chemosynapse [29], along with β-arrestin, AP-2, PP2A, and HIP [17]–[19] that bind CXCR2 upon stimulation with CXCL8 and regulate CXCR2-mediated polarization and chemotaxis.

In addition to CXCR2, other related CXC chemokine receptors (CXCR1, CXCR3 and CXCR4 CTDs) bind to LASP-1 probably through their LKIL motif. The LKIL motif was observed to be critical for CXCR2 mediated chemotaxis [30], [48]. Through interaction with these chemokine receptors, LASP-1 may also regulate tumor cell migration or possibly survival. Interestingly, these chemokine receptors, with the exception of CXCR1, have been shown to play a role in metastasis of mammary carcinoma. Additionally, CXCR2 and CXCR4 can recruit Gr-1+CD11b+ myeloid cells into invasive front of mammary tumor, an event that directly promotes tumor metastasis. Elevated expression of CXCR3 in human mammary carcinoma correlates with poor survival [49]–[51]. LASP-1 regulates cell migration in 293-CXCR2 cells (in this study), mammary tumor [27] and ovarian tumor [28] cell lines and MEFS [47]. By optimizing the tumor cell motility towards cytokines (including chemokines) and growth factors in the tumor microenvironment (i.e., not random migration), LASP-1 is likely is an important mediator of tumor growth and invasion.

The differential direct interaction of CXCR2-CTD and CXCR4-CTD with non-phosphorylated and phosphorylated LASP-1 is intriguing. The differential affinity of different CXC chemokine receptors for LASP-1 dependent upon LASP-1 phosphorylation likely has different functional consequences, allowing for a fine tuning of the biological responses to chemokines. Protein kinase A (PKA) and protein kinase G (PKG) have been implicated in the phosphorylation of LASP-1 at S146 in gastric parietal cells and in human platelets respectively [36], [41]. CXCR2 and CXCR4, being Gα_i_-coupled receptors, can only indirectly activate PKA through a second round of G-protein signaling. Alternatively, α4β1 integrins at the leading edge are reported to anchor and activate type I PKA leading to polarized activation of PKA at the leading edge. This adhesion mediated localized PKA activation event could potentially phosphorylate LASP-1 at the nascent focal adhesions and anchored to the branched actin network at the leading edge [52], [53]. This scenario might be related to dHL-60 cells since these were seeded onto fibronectin, a specific ligand for α4β1. Another report implies that a phosphomimetic LASP-1 mutant (S146D) reduces the affinity for F-actin leading to predominant cytosolic re-localization and reduced migration in transfected Ptk2 cells [36]. The exact role of LASP-1 phosphorylation at S146 in cell migration remains to be understood.

The site-directed mutagenesis approach used in this study identified amino acid residues I323-L324 of CXCR2-CTD as critical determinants for the binding of LIM domain of LASP-1, along with some flanking residues. Since this same mutant of CXCR2 has been shown earlier to be deficient in binding to AP-2 [19] and Hsp70 interacting protein (HIP) [18], we conclude that the I323-L324 residues of CXCR2 are required for CXCR2 binding to AP-2, HIP and LASP-1 in a spatiotemporal manner resulting in efficient and controlled chemotaxis.

Dominant negative LIM-LIM-CAAX not only impaired CXCR2 mediated chemotaxis but also affected the activation of many proteins involved in focal adhesion turnover and cell motility. There were remarkable deficiencies in CXCL8 induced tyrosine phosphorylation and activation of *c*-*src*, paxillin, p130CAS and activation of PAK1 and ERK1/2 when the interaction of CXCR2 with LASP-1 was interrupted by the expression of the LIM-LIM-CAAX. Previously, we have shown that ligand activated CXCR2 can induce phosphorylation of p130CAS and PAK1 and that PAK1 was required for CXCL1-induced chemotaxis [35], [45]. PAK1 is suggested to regulate lamellipodial dynamics via ERK1/2 in mouse macrophages. PAK1 is also very important in controlling cofilin activity (an actin severing protein) through LIM kinase 1 (LIMK1). Thus, deficient PAK1 and ERK1/2 activation in LIM-LIM-CAAX cells might affect the lamellipodial dynamics. Interestingly, active p130 CAS, paxillin and ERK have been implied to be critical for the nascent adhesion turnover at the leading edge, a process that is key to the cell migration [12]. Paxillin phosphorylation at tyrosines 118 and 31 has been reported to promote migration [54]. Also, Y31F and Y118F-paxillin when transduced into paxillin null cells, the rate constants for the adhesion disassembly decreased indicating that phosphorylation of Y31 and Y118 promotes paxillin disassembly from the nascent adhesions [12]. Conceivably, deficient paxillin phosphorylation and activation in both LIM-LIM-CAAX cells and LASP knock down (LASP-KD5) cells might affect the maturation or turnover of nascent adhesions near the leading edge of the cell. In addition, paxillin has been shown to influence microtubule catastrophes at focal adhesion sites. The phosphorylation of paxillin promotes its disassembly from the focal adhesions and hence might influence microtubule dynamics and hence the delivery of proteins necessary for cell migration by its presence or absence at the adhesion sites [55], [56]. Active ERK1/2 kinase phosphorylation of the myosin light chain kinase (MLCK) has been implicated in acto-myosin assembly and contractility and the mechanical stretching reportedly activates talin and p130CAS [57], [58].

The observed compromise of CXCL8 induced chemotaxis in LASP-KD5 cells was due to an increase in basal migration and a lack of corresponding increase in chemotactic migration upon incubation with CXCL8. This contributed to a relatively flat chemotactic profile than the bell-shaped curve observed with non-silenced cells. The observed increase in basal migration of LASP-KD5 cells was supported by the observed basal activation of *c-Src*, p130CAS, PAK1. Interestingly, in LASP-1 knock out mouse embryo fibroblasts, reported elaboration of several chemokines including CXCL1 might also occur in LASP-KD5 cells [47]. This scenario could operate in LASP-KD5 cells that would explain the basal activation of several proteins including *c-Src*, p130CAS, PAK1. Alternatively, LASP-2 might substitute for the knock down of LASP-1 protein in increasing the basal migration. But the NCBI database analysis for LASP-2 expression in human embryonic kidney-293 (HEK-293) cells revealed that LASP-2 expression is absent in these cells (Ref. # GDS916). In addition to regulating the CXCR2-mediated chemotaxis, knock down of LASP-1 may also affect the molecular profile of the proteins present at the focal adhesions. We detected an increase in total p130CAS level (without a net increase in pY249-p130CAS level) in LASP-KD5 cells. This may result from a compensatory up-regulation as a similar increase was observed upon knock down of FAK [59]. These subtle events may be associated with the changes in basal migration (in the absence of CXCL8) as well as CXCL8 induced chemotaxis.

In several of the experiments described here, we observed different effects on signaling when CXCR2 interaction with LASP-1 was interrupted by expression of LIM-LIM-CAAX, as opposed to knock down of LASP-1. As the LIM domain is conserved between LASP-1 and LASP-2, LIM-LIM-CAAX operated effectively as a dominant negative for both of the proteins. Since knocking down LASP-1 effectively interrupts almost all LASP-1 protein-protein interactions, this is not an unexpected finding. Indeed, similar findings have been reported when LASP-1 was knocked out with an increase in LPP [47] and up regulation of p130CAS with the FAK knock down [59]. Also, pyruvate kinase, 14-3-3, Hsp27 were up regulated and enolase-1, glucose dehydrogenase were down regulated with the knock down of LASP-1 in SKOV-3 cells [28]. Alterations in the protein level of metabolic enzymes, adaptor proteins and heat shock proteins upon LASP-1 knock down may alter the cellular dynamics or motility. Similarly, if LASP-1 interacting protein zyxin is deleted, it leads to loss of Mena and VASP from focal adhesions [60]. Based on these studies, it is possible that knock down of proteins that reside at focal adhesions probably may lead to incomplete or improper assembly of the network of proteins found at the focal adhesions. In contrast, the LIM-LIM-CAAX should only interrupt CXCR2/LASP-1 or LASP-2 interactions at the membrane. While additional proteins may also bind to LIM domain of LASP-1 at the plasma membrane, these interactions are yet to be described. We have demonstrated for the first time that CXCR2 directly interacts with LASP-1 and this interaction is necessary for optimal ligand mediated chemotaxis through CXCR2. CXCR2 also becomes the first known protein to bind to the LIM domain of LASP-1 thus providing a function for its LIM domain. The data in this study provide a basis for potential regulation of CXCR2 mediated directed migration of leukocytes and endothelial cells by LASP-1.

## Supporting Information

Figure S1 Analysis of Rap1 activation and β1-integrin levels in LIM-LIM-CAAX and LASP-KD5 cells. A. Differential Rap1 activation profile in LIM-LIM-CAAX cells. Vector and LIM-LIM-CAAX transfected cells plated on collagen IV were stimulated with CXCL8 (50 ng/mL) for different time points. Lysates (1.5 mg) were incubated with 100 µg of GST-RBD-Ral-GDS and active Rap1 (Rap1-GTP) was isolated. Bound Rap1-GTP was separated on 15% SDS-PAGE and analyzed by western blotting. Total Rap1 from lysates was used as the loading control. Rap1-GTP for the vector control at 0 min time point is normalized to 1 and any increase or decrease in the Rap1-GTP level is indicated. B. Differential Rap1 activation profile in LASP-KD5 cells. Control silenced and LASP-KD5 cells were plated on collagen IV and stimulated with CXCL8 at 50 ng/mL for the indicated time points and lysates were prepared. Lysates (1.5 mg) were incubated with 100 µg of GST-RBD-Ral-GDS and Rap1-GTP was isolated. Bound Rap1-GTP was separated on 15% SDS-PAGE and analyzed by western blotting. Total Rap1 from lysates was used as the loading control. Rap1-GTP for the non-silencing control at 0 min time point is normalized to 1 and any increase or decrease in the Rap1-GTP level is indicated. C. β1-integrin levels remain unchanged upon expression of the LIM-LIM-CAAX protein in 293-CXCR2 cells. 293-CXCR2 cells were transiently transfected with vector (pcDNA3.0) or HA-tagged LIM-LIM-CAAX cDNA. Transfected cells were plated on collagen IV coated dishes 48 h post transfection and were stimulated with CXCL8 at 50 ng/mL for the indicated time points and lysates were prepared. 50 µg of total protein lysates was separated by 10% SDS-PAGE and blotted for any change in endogenous β1-integrin level. V - Vector control; L - LIM-LIM-CAAX. D. β1-integrin levels remain unchanged in LASP-KD5 293-CXCR2 cells. Control silenced and LASP-KD5 cells were plated on collagen IV and stimulated with CXCL8 at 50 ng/mL for the indicated time points and lysates were prepared. 50 µg of total protein lysates was separated by 10% SDS-PAGE and blotted for any change in endogenous β1-integrin level.
(0.19 MB TIF)

Figure S2 Levels of lipoma prefrerred protein (LPP) and zyxin remain unchanged in LASP-KD5 cells. Control silenced and LASP-KD5 cells were plated on collagen IV and stimulated with CXCL8 at 50 ng/mL for the indicated time points and lysates were prepared. 50 µg of total protein lysates was separated by 10% SDS-PAGE and blotted for any change in the level of proteins related to LASP-1.
(0.12 MB TIF)

Figure S3 Analysis of co-distribution of CXCR2 and endogenous LASP-1 in CXCL8 polarized dHL-60 cells differentiated along the neutrophil lineage. A. Line scan analysis of CXCL8 stimulated dHL-60 cells at 1 min (top) and 2 min (bottom). The confocal image is shown on the left and the corresponding line scan distribution profile of fluorescence intensities of CXCR2, LASP-1 and PH-Akt-GFP obtained by metamorph analysis is shown on the right. The distribution profile based on the pixels (intensity of the fluorescence) starts from the rear to the front of the cell. Red trace - LASP-1; Green trace - PH-Akt-GFP; Blue trace - CXCR2. B. Line scan analysis of CXCL8 polarized dHL-60 cells in Zigmond chamber. The confocal images of three different dHL60 cells polarized in the Zigmond chamber is depicted on the left and the corresponding line scan distribution profile obtained by metamorph analysis is displayed on the right. The distribution profile based on the intensity of the fluorescence starts from the rear to the front of the cell. Red trace - F-actin; Green trace - CXCR2; Blue trace - LASP-1.
(0.50 MB TIF)

## References

[1] Addison CL, Daniel TO, Burdick MD, Liu H, Ehlert JE (2000). The CXC chemokine receptor 2, CXCR2, is the putative receptor for ELR+ CXC chemokine-induced angiogenic activity.. J Immunol.

[2] Devalaraja RM, Nanney LB, Du J, Qian Q, Yu Y (2000). Delayed wound healing in CXCR2 knockout mice.. J Invest Dermatol.

[3] Horton LW, Yu Y, Zaja-Milatovic S, Strieter RM, Richmond A (2007). Opposing roles of murine duffy antigen receptor for chemokine and murine CXC chemokine receptor-2 receptors in murine melanoma tumor growth.. Cancer Res.

[4] Raman D, Baugher PJ, Thu YM, Richmond A (2007). Role of chemokines in tumor growth.. Cancer Lett.

[5] Yanagawa J, Walser TC, Zhu LX, Hong L, Fishbein MC (2009). Snail promotes CXCR2 ligand-dependent tumor progression in non-small cell lung carcinoma.. Clin Cancer Res.

[6] Matsuo Y, Raimondo M, Woodward TA, Wallace MB, Gill KR (2009). CXC-chemokine/CXCR2 biological axis promotes angiogenesis in vitro and in vivo in pancreatic cancer.. Int J Cancer.

[7] Strieter RM, Burdick MD, Mestas J, Gomperts B, Keane MP (2006). Cancer CXC chemokine networks and tumour angiogenesis.. Eur J Cancer.

[8] Strieter RM, Burdick MD, Gomperts BN, Belperio JA, Keane MP (2005). CXC chemokines in angiogenesis.. Cytokine Growth Factor Rev.

[9] Parent CA, Devreotes PN (1999). A cell's sense of direction.. Science.

[10] Petrie RJ, Doyle AD, Yamada KM (2009). Random versus directionally persistent cell migration.. Nat Rev Mol Cell Biol.

[11] Webb DJ, Parsons JT, Horwitz AF (2002). Adhesion assembly, disassembly and turnover in migrating cells – over and over and over again.. Nat Cell Biol.

[12] Webb DJ, Donais K, Whitmore LA, Thomas SM, Turner CE (2004). FAK-Src signalling through paxillin, ERK and MLCK regulates adhesion disassembly.. Nat Cell Biol.

[13] Deakin NO, Turner CE (2008). Paxillin comes of age.. J Cell Sci.

[14] Lauffenburger DA, Horwitz AF (1996). Cell migration: a physically integrated molecular process.. Cell.

[15] Neel NF (2008). Regulation of CXC chemokine receptor function through intracellular trafficking and novel receptor-interacting proteins.. Ph D Dissertation, Vanderbilt University.

[16] Raman D, Neel NF, Sai J, Mernaugh RL, Ham AJ (2009). Characterization of chemokine receptor CXCR2 interacting proteins using a proteomics approach to define the CXCR2 “chemosynapse”.. Methods Enzymol.

[17] Fan GH, Yang W, Sai J, Richmond A (2001). Phosphorylation-independent association of CXCR2 with the protein phosphatase 2A core enzyme.. J Biol Chem.

[18] Fan GH, Yang W, Sai J, Richmond A (2002). Hsc/Hsp70 interacting protein (hip) associates with CXCR2 and regulates the receptor signaling and trafficking.. J Biol Chem.

[19] Fan GH, Yang W, Wang XJ, Qian Q, Richmond A (2001). Identification of a motif in the carboxyl terminus of CXCR2 that is involved in adaptin 2 binding and receptor internalization.. Biochemistry.

[20] Tomasetto C, Moog-Lutz C, Regnier CH, Schreiber V, Basset P (1995). Lasp-1 (MLN 50) defines a new LIM protein subfamily characterized by the association of LIM and SH3 domains.. FEBS Lett.

[21] Schmeichel KL, Beckerle MC (1994). The LIM domain is a modular protein-binding interface.. Cell.

[22] Chew CS, Chen X, Parente JA, Tarrer S, Okamoto C (2002). Lasp-1 binds to non-muscle F-actin in vitro and is localized within multiple sites of dynamic actin assembly in vivo.. J Cell Sci.

[23] Rachlin AS, Otey CA (2006). Identification of palladin isoforms and characterization of an isoform-specific interaction between Lasp-1 and palladin.. J Cell Sci.

[24] Lin YH, Park ZY, Lin D, Brahmbhatt AA, Rio MC (2004). Regulation of cell migration and survival by focal adhesion targeting of Lasp-1.. J Cell Biol.

[25] Spence HJ, McGarry L, Chew CS, Carragher NO, Scott-Carragher LA (2006). AP-1 differentially expressed proteins Krp1 and fibronectin cooperatively enhance Rho-ROCK-independent mesenchymal invasion by altering the function, localization, and activity of nondifferentially expressed proteins.. Mol Cell Biol.

[26] Grunewald TG, Butt E (2008). The LIM and SH3 domain protein family: structural proteins or signal transducers or both?. Mol Cancer.

[27] Grunewald TG, Kammerer U, Schulze E, Schindler D, Honig A (2006). Silencing of LASP-1 influences zyxin localization, inhibits proliferation and reduces migration in breast cancer cells.. Exp Cell Res.

[28] Grunewald TG, Kammerer U, Winkler C, Schindler D, Sickmann A (2007). Overexpression of LASP-1 mediates migration and proliferation of human ovarian cancer cells and influences zyxin localisation.. Br J Cancer.

[29] Neel NF, Barzik M, Raman D, Sobolik-Delmaire T, Sai J (2009). VASP is a CXCR2-interacting protein that regulates CXCR2-mediated polarization and chemotaxis.. J Cell Sci.

[30] Sai J, Walker G, Wikswo J, Richmond A (2006). The IL sequence in the LLKIL motif in CXCR2 is required for full ligand-induced activation of Erk, Akt, and chemotaxis in HL60 cells.. J Biol Chem.

[31] Baugher PJ, Richmond A (2008). The carboxyl-terminal PDZ ligand motif of chemokine receptor CXCR2 modulates post-endocytic sorting and cellular chemotaxis.. J Biol Chem.

[32] Mueller SG, Schraw WP, Richmond A (1994). Melanoma growth stimulatory activity enhances the phosphorylation of the class II interleukin-8 receptor in non-hematopoietic cells.. J Biol Chem.

[33] Marchese A, Benovic JL (2001). Agonist-promoted ubiquitination of the G protein-coupled receptor CXCR4 mediates lysosomal sorting.. J Biol Chem.

[34] Sai J, Raman D, Liu Y, Wikswo J, Richmond A (2008). Parallel phosphatidylinositol 3-kinase (PI3K)-dependent and Src-dependent pathways lead to CXCL8-mediated Rac2 activation and chemotaxis.. J Biol Chem.

[35] Wang D, Sai J, Carter G, Sachpatzidis A, Lolis E (2002). PAK1 kinase is required for CXCL1-induced chemotaxis.. Biochemistry.

[36] Butt E, Gambaryan S, Gottfert N, Galler A, Marcus K (2003). Actin binding of human LIM and SH3 protein is regulated by cGMP- and cAMP-dependent protein kinase phosphorylation on serine 146.. J Biol Chem.

[37] Van Keymeulen A, Wong K, Knight ZA, Govaerts C, Hahn KM (2006). To stabilize neutrophil polarity, PIP3 and Cdc42 augment RhoA activity at the back as well as signals at the front.. J Cell Biol.

[38] Stephens L, Ellson C, Hawkins P (2002). Roles of PI3Ks in leukocyte chemotaxis and phagocytosis.. Curr Opin Cell Biol.

[39] Weiner OD (2002). Regulation of cell polarity during eukaryotic chemotaxis: the chemotactic compass.. Curr Opin Cell Biol.

[40] Chew CS, Parente JA, Chen X, Chaponnier C, Cameron RS (2000). The LIM and SH3 domain-containing protein, lasp-1, may link the cAMP signaling pathway with dynamic membrane restructuring activities in ion transporting epithelia.. J Cell Sci.

[41] Chew CS, Parente JA, Zhou C, Baranco E, Chen X (1998). Lasp-1 is a regulated phosphoprotein within the cAMP signaling pathway in the gastric parietal cell.. Am J Physiol.

[42] Li B, Zhuang L, Trueb B (2004). Zyxin interacts with the SH3 domains of the cytoskeletal proteins LIM-nebulette and Lasp-1.. J Biol Chem.

[43] Klemke RL, Leng J, Molander R, Brooks PC, Vuori K (1998). CAS/Crk coupling serves as a “molecular switch” for induction of cell migration.. J Cell Biol.

[44] Cho SY, Klemke RL (2002). Purification of pseudopodia from polarized cells reveals redistribution and activation of Rac through assembly of a CAS/Crk scaffold.. J Cell Biol.

[45] Schraw W, Richmond A (1995). Melanoma growth stimulatory activity signaling through the class II interleukin-8 receptor enhances the tyrosine phosphorylation of Crk-associated substrate, p130, and a 70-kilodalton protein.. Biochemistry.

[46] Cheresh DA, Leng J, Klemke RL (1999). Regulation of cell contraction and membrane ruffling by distinct signals in migratory cells.. J Cell Biol.

[47] Zhang H, Chen X, Bollag WB, Bollag RJ, Sheehan DJ (2009). Lasp1 gene disruption is linked to enhanced cell migration and tumor formation.. Physiol Genomics.

[48] Sai J, Fan GH, Wang D, Richmond A (2004). The C-terminal domain LLKIL motif of CXCR2 is required for ligand-mediated polarization of early signals during chemotaxis.. J Cell Sci.

[49] Fulton AM (2009). The chemokine receptors CXCR4 and CXCR3 in cancer.. Curr Oncol Rep.

[50] Ma X, Norsworthy K, Kundu N, Rodgers WH, Gimotty PA (2009). CXCR3 expression is associated with poor survival in breast cancer and promotes metastasis in a murine model.. Mol Cancer Ther.

[51] Yang L, Huang J, Ren X, Gorska AE, Chytil A (2008). Abrogation of TGF beta signaling in mammary carcinomas recruits Gr-1+CD11b+ myeloid cells that promote metastasis.. Cancer Cell.

[52] Lim CJ, Han J, Yousefi N, Ma Y, Amieux PS (2007). Alpha4 integrins are type I cAMP-dependent protein kinase-anchoring proteins.. Nat Cell Biol.

[53] Lim CJ, Kain KH, Tkachenko E, Goldfinger LE, Gutierrez E (2008). Integrin-mediated protein kinase A activation at the leading edge of migrating cells.. Mol Biol Cell.

[54] Petit V, Boyer B, Lentz D, Turner CE, Thiery JP (2000). Phosphorylation of tyrosine residues 31 and 118 on paxillin regulates cell migration through an association with CRK in NBT-II cells.. J Cell Biol.

[55] Efimov A, Schiefermeier N, Grigoriev I, Ohi R, Brown MC (2008). Paxillin-dependent stimulation of microtubule catastrophes at focal adhesion sites.. J Cell Sci.

[56] Siesser PM, Meenderink LM, Ryzhova L, Michael KE, Dumbauld DW (2008). A FAK/Src chimera with gain-of-function properties promotes formation of large peripheral adhesions associated with dynamic actin assembly.. Cell Motil Cytoskeleton.

[57] Alexander NR, Branch KM, Parekh A, Clark ES, Iwueke IC (2008). Extracellular matrix rigidity promotes invadopodia activity.. Curr Biol.

[58] Parekh A, Weaver AM (2009). Regulation of cancer invasiveness by the physical extracellular matrix environment.. Cell Adh Migr.

[59] Wendt MK, Smith JA, Schiemann WP (2009). p130Cas is required for mammary tumor growth and transforming growth factor-beta-mediated metastasis through regulation of Smad2/3 activity.. J Biol Chem.

[60] Hoffman LM, Jensen CC, Kloeker S, Wang CL, Yoshigi M (2006). Genetic ablation of zyxin causes Mena/VASP mislocalization, increased motility, and deficits in actin remodeling.. J Cell Biol.

