# Supplementary figures and images for: LIM and SH3 Protein -1 Modulates CXCR2-Mediated Cell Migration
Source: PLoS One. 2010 Apr 19;5(4):e10050. doi: 10.1371/journal.pone.0010050 (PMC2856662; doi:10.1371/journal.pone.0010050)

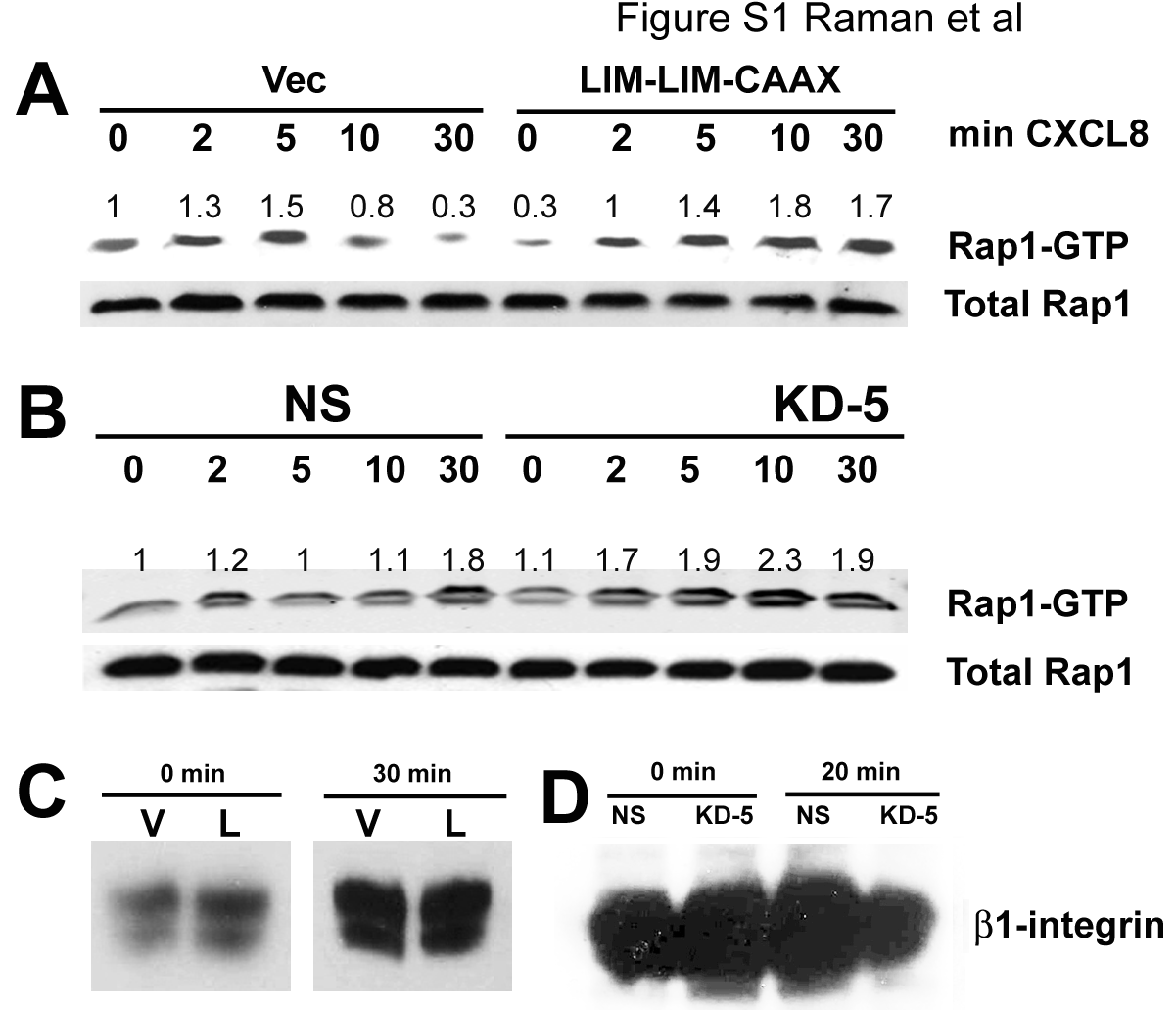

Supplement: Figure S1 — Analysis of Rap1 activation and β1-integrin levels in LIM-LIM-CAAX and LASP-KD5 cells. A. Differential Rap1 activation profile in LIM-LIM-CAAX cells. Vector and LIM-LIM-CAAX transfected cells plated on collagen IV were stimulated with CXCL8 (50 ng/mL) for different time points. Lysates (1.5 mg) were incubated with 100 µg of GST-RBD-Ral-GDS and active Rap1 (Rap1-GTP) was isolated. Bound Rap1-GTP was separated on 15% SDS-PAGE and analyzed by western blotting. Total Rap1 from lysates was used as the loading control. Rap1-GTP for the vector control at 0 min time point is normalized to 1 and any increase or decrease in the Rap1-GTP level is indicated. B. Differential Rap1 activation profile in LASP-KD5 cells. Control silenced and LASP-KD5 cells were plated on collagen IV and stimulated with CXCL8 at 50 ng/mL for the indicated time points and lysates were prepared. Lysates (1.5 mg) were incubated with 100 µg of GST-RBD-Ral-GDS and Rap1-GTP was isolated. Bound Rap1-GTP was separated on 15% SDS-PAGE and analyzed by western blotting. Total Rap1 from lysates was used as the loading control. Rap1-GTP for the non-silencing control at 0 min time point is normalized to 1 and any increase or decrease in the Rap1-GTP level is indicated. C. β1-integrin levels remain unchanged upon expression of the LIM-LIM-CAAX protein in 293-CXCR2 cells. 293-CXCR2 cells were transiently transfected with vector (pcDNA3.0) or HA-tagged LIM-LIM-CAAX cDNA. Transfected cells were plated on collagen IV coated dishes 48 h post transfection and were stimulated with CXCL8 at 50 ng/mL for the indicated time points and lysates were prepared. 50 µg of total protein lysates was separated by 10% SDS-PAGE and blotted for any change in endogenous β1-integrin level. V - Vector control; L - LIM-LIM-CAAX. D. β1-integrin levels remain unchanged in LASP-KD5 293-CXCR2 cells. Control silenced and LASP-KD5 cells were plated on collagen IV and stimulated with CXCL8 at 50 ng/mL for the indicated time points and lysates were [file pone.0010050.s001.tif]

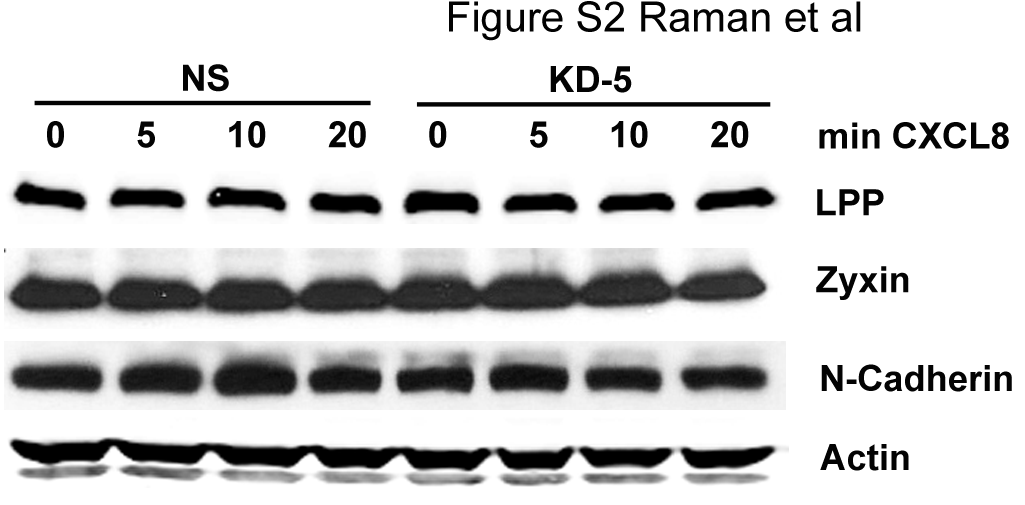

Supplement: Figure S2 — Levels of lipoma prefrerred protein (LPP) and zyxin remain unchanged in LASP-KD5 cells. Control silenced and LASP-KD5 cells were plated on collagen IV and stimulated with CXCL8 at 50 ng/mL for the indicated time points and lysates were prepared. 50 µg of total protein lysates was separated by 10% SDS-PAGE and blotted for any change in the level of proteins related to LASP-1. (0.12 MB TIF) [file pone.0010050.s002.tif]

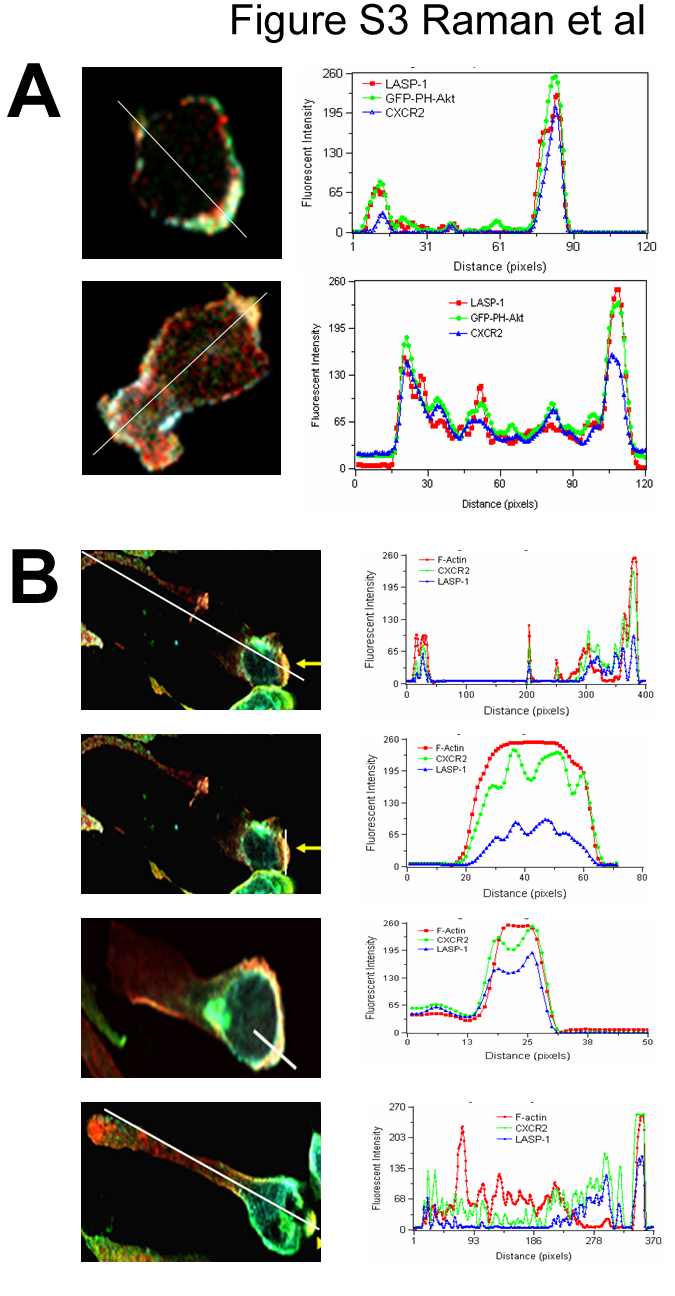

Supplement: Figure S3 — Analysis of co-distribution of CXCR2 and endogenous LASP-1 in CXCL8 polarized dHL-60 cells differentiated along the neutrophil lineage. A. Line scan analysis of CXCL8 stimulated dHL-60 cells at 1 min (top) and 2 min (bottom). The confocal image is shown on the left and the corresponding line scan distribution profile of fluorescence intensities of CXCR2, LASP-1 and PH-Akt-GFP obtained by metamorph analysis is shown on the right. The distribution profile based on the pixels (intensity of the fluorescence) starts from the rear to the front of the cell. Red trace - LASP-1; Green trace - PH-Akt-GFP; Blue trace - CXCR2. B. Line scan analysis of CXCL8 polarized dHL-60 cells in Zigmond chamber. The confocal images of three different dHL60 cells polarized in the Zigmond chamber is depicted on the left and the corresponding line scan distribution profile obtained by metamorph analysis is displayed on the right. The distribution profile based on the intensity of the fluorescence starts from the rear to the front of the cell. Red trace - F-actin; Green trace - CXCR2; Blue trace - LASP-1. (0.50 MB TIF) [file pone.0010050.s003.tif]
